# Therapeutic potential of MSCs and their exosomes in hepatic Ischaemia-Reperfusion injury: a systematic review and meta-analysis of rodent studies

**DOI:** 10.1093/stcltm/szaf078

**Published:** 2026-01-26

**Authors:** Yanxi Mu, Weixiong Zhu, Wentao Ma, Yu Cheng, Bo Ren, Yusheng Cheng, Wence Zhou

**Affiliations:** The Second Clinical Medical College, Lanzhou University, Lanzhou, 730000, China; The Second Clinical Medical College, Lanzhou University, Lanzhou, 730000, China; The Second Clinical Medical College, Lanzhou University, Lanzhou, 730000, China; The Second Clinical Medical College, Lanzhou University, Lanzhou, 730000, China; The Second Clinical Medical College, Lanzhou University, Lanzhou, 730000, China; The Second Clinical Medical College, Lanzhou University, Lanzhou, 730000, China; Department of General Surgery, Biotherapy, Lanzhou University Second Hospital, Lanzhou, 730000, China; Gansu Province Precision Diagnosis and Treatment Engineering Research Center of Hepatobiliary Pancreatic Diseases, Gansu Province Key Laboratory of Environmental Oncology, Lanzhou, 730000, China; The Second Clinical Medical College, Lanzhou University, Lanzhou, 730000, China; Department of General Surgery, Biotherapy, Lanzhou University Second Hospital, Lanzhou, 730000, China; Gansu Province Precision Diagnosis and Treatment Engineering Research Center of Hepatobiliary Pancreatic Diseases, Gansu Province Key Laboratory of Environmental Oncology, Lanzhou, 730000, China

**Keywords:** Mesenchymal stem cells (MSCs), exosomes, Hepatic Ischaemia-Reperfusion Injury (HIRI), rodent models, meta-analysis

## Abstract

**Objective:**

This meta-analysis comprehensively evaluates the therapeutic efficacy and mechanisms of mesenchymal stem cells (MSCs) and their exosomes in rodent models of hepatic ischemia-reperfusion injury (HIRI), providing preclinical support for future clinical translation.

**Methods:**

In accordance with the PRISMA guidelines, we systematically searched PubMed, Web of Science, Embase, Cochrane Library, and ClinicalTrials.gov for studies published from inception to January 13, 2025, and identified 64 eligible studies. Risk of bias was evaluated using the SYRCLE tool, and Review Manager 5.4.1 was employed for meta-analysis, calculating SMD and 95%CI. Primary outcomes included liver function (ALT/AST), histopathological scores (Suzuki’s score, necrotic area ratio), inflammatory cytokines (TNF-α), and apoptosis markers (c-caspase 3).

**Results:**

MSCs and their exosomes significantly ameliorated HIRI. In the 60-minute ischemia group, ALT (SMD = 3.49, *P <* .00001) and AST (SMD = 3.86, *P <* .00001) decreased, along with lower Suzuki scores (SMD = 3.12), necrotic area ratios (SMD = 3.56), and TNF-α levels (SMD = 2.83). In the 90-minute group, ALT (SMD = 4.09, *P <* .00001) and AST (SMD = 3.78, *P <* .00001) were also reduced. Mechanistically, MSCs exert therapeutic effects through antioxidative, anti-inflammatory, anti-apoptotic, and pro-regenerative pathways. Considerable heterogeneity (*I*^2^ = 52–86%) was observed, likely due to variations in dosage (1 × 10^5^-1 × 10^9^ cells), administration routes (intravenous/portal vein), and reperfusion durations (3–24 hours). Genetic modifications (e.g., HO-1 overexpression) further enhanced therapeutic outcomes.

**Conclusion:**

MSCs and their exosomes mitigate HIRI through multi-target mechanisms but requires standardized protocols. Future studies should prioritize large-animal validation and translational research to facilitate precision clinical application.

## Graphical abstract

**Graphical Abstract:**
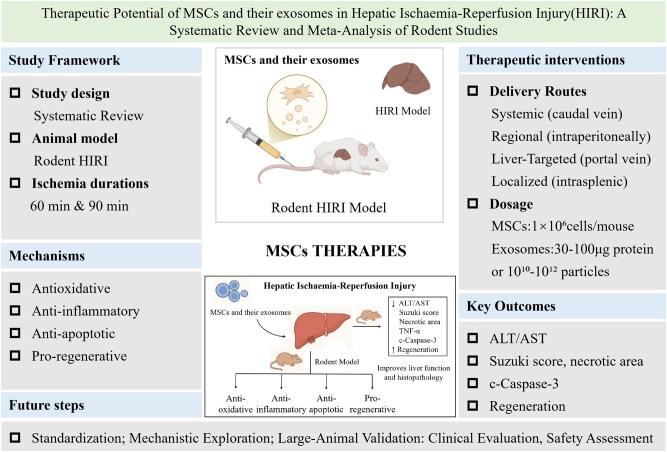

Significance Statement Hepatic ischemia-reperfusion injury (HIRI) lacks effective therapy. This meta-analysis demonstrates that mesenchymal stem cells (MSCs) and their exosomes markedly alleviate HIRI in rodent models by improving liver function and reducing inflammation, oxidative stress, and apoptosis. These findings provide strong preclinical evidence supporting MSCs-based interventions as promising candidates for translational application in hepatic surgery and transplantation.

## Introduction

Hepatic ischemia-reperfusion injury (HIRI) is a critical clinical challenge in liver surgery, frequently encountered in hepatectomy, liver transplantation, and hemorrhagic shock.^1^ HIRI is a major risk factor for primary graft dysfunction or failure and a key trigger for acute and chronic rejection.^1^ Hypoxia-induced HIRI involves an exogenous antigen-dependent local inflammatory response.^2^ Persistent HIRI not only causes hepatocyte apoptosis and necrosis, which can result in severe liver failure, but also provokes a substantial release of inflammatory mediators, thereby inducing systemic inflammatory response syndrome (SIRS). This further leads to multiple organ dysfunction syndrome (MODS), and may ultimately progress to multiple organ failure, significantly increasing the mortality rate of patients.

In recent years, stem cell-based therapies have emerged as a prominent strategy for mitigating HIRI, owing to their unique capacities for tissue repair and immunomodulation. Compared with conventional pharmacologic interventions, stem cell therapy offers multi-target and durable therapeutic benefits with an established safety profile in clinical applications. Among various stem cell populations, mesenchymal stem cells (MSCs) are regarded as the most clinically translatable cell type because of their broad tissue availability, ease of isolation, and robust immunoregulatory properties. MSCs reside in multiple mesenchymal tissues including bone marrow, adipose tissue, umbilical cord, and dental tissues and are characterized by adherence to plastic, expression of CD73, CD90, and CD105, lack of CD34, CD45, and HLA-DR expression, and tri-lineage differentiation potential toward osteogenic, chondrogenic, and adipogenic fates.^3^ Increasing evidence further indicates that the therapeutic actions of MSCs are predominantly mediated by their rich paracrine activity rather than by direct differentiation. MSCs-derived cytokines, chemokines, and extracellular vesicles, particularly exosomes, can reprogram innate and adaptive immune responses, attenuate excessive inflammation, and promote tissue repair and regeneration.^3^^,^^4^ Notably, MSCs derived from different tissues display substantial heterogeneity in proliferative potential, secretory profiles, and immunomodulatory potency; these variations are shaped by microenvironmental cues, inflammatory status, and metabolic reprogramming.^5^

Within the context of HIRI, MSCs-based therapy has gained considerable attention due to its pronounced immunomodulatory and regenerative capacities. Accumulating preclinical evidence demonstrates that MSCs rapidly home to the injured liver and ameliorate tissue damage by suppressing proinflammatory cytokine release, attenuating oxidative stress and apoptosis, and restoring microenvironmental homeostasis.^6^^,^^7^ Importantly, these therapeutic benefits also rely heavily on MSCs-derived paracrine mediators, including exosomes and diverse soluble factors that orchestrate immune regulation and tissue protection.^8^ Despite promising outcomes, substantial variation remains across studies regarding cell dose, timing, and route of administration, underscoring the need for greater standardization to optimize clinical translation.

This study aims to systematically review and meta-analyze existing rodent experimental evidence to evaluate the pooled effects of MSCs and their exosomes on liver function (ALT/AST) and histopathological outcomes across different ischemiareperfusion time points, and to explore potential sources of interstudy heterogeneity. The findings are expected to provide evidence-based insights to inform the design of future clinical trials and to facilitate the translation of stem cell therapy from preclinical research to precision medicine.

## Materials and methods

### Study selection

The inclusion criteria for the study are as follows: (1) In vivo rodent models (mice or rats); (2) The experimental group receive stem cell therapy; (3) The primary outcome index: HIRI; (4) The research was published in English. The exclusion criteria for the study are as follows: (1) There was no control group in the study; (2) Type of articles: Case reports, conference abstracts, posters, reviews, meta-analyses and letters from readers; (3) Lack of the full text; (4) No result or incomplete data; (5) Duplicate records. In this study, two researchers independently screened the titles, abstracts, and full texts, with any disagreements resolved through consensus discussion involving a third reviewer.

### Protocol and registration

**Figure 1.**
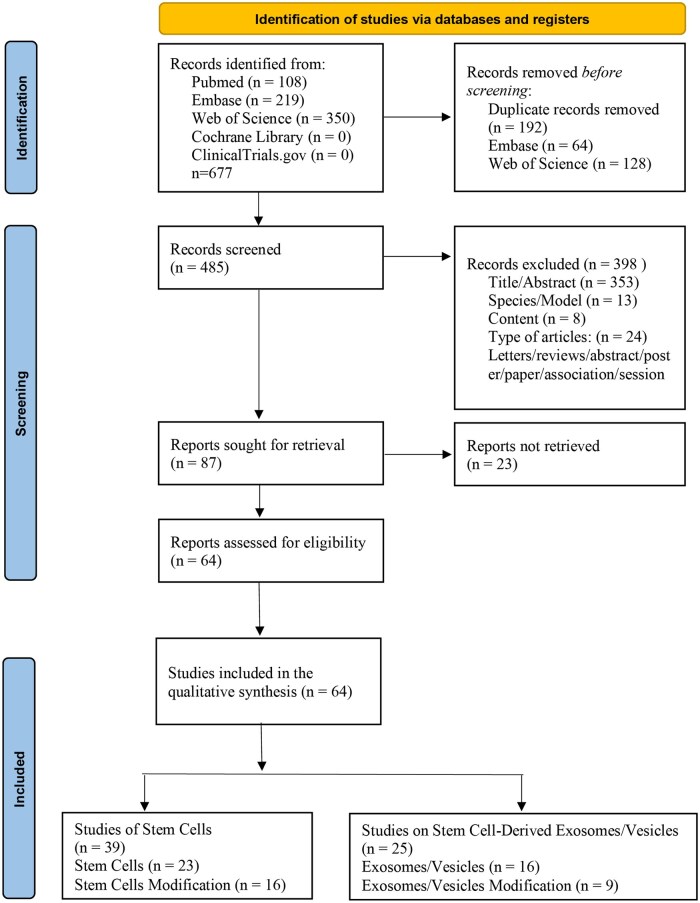
Prisma flow diagram. Search strategy and selection process of the included studies.

This study adhered to the methodological standards established by the EQUATOR Network (Enhancing the Quality and Transparency of Health Research), and reported according to PRISMA guidelines (Figure 1). The research protocol was prospectively registered in the PROSPERO international prospective register of systematic reviews (registration number: CRD420250638552).

### Search strategy

This study adopted a systematic literature retrieval method, and cross-database retrieval was independently carried out by two researchers. The search scope includes the following core biomedical databases: PubMed, Web of Science, Embase, Cochrane Library, and ClinicalTrials.gov. Search strategies followed the PICOS framework using the following keywords: (1) “hepatic ischemia-reperfusion injury” OR “liver IRI” OR “HIRI”; (2) “stem cells” OR “mesenchymal stromal cells” OR “MSCs”; (3) “murine” OR “rodent” OR “animal model. The search was conducted from database inception to January 13, 2025, placing particular emphasis on studies published after January 2020. The complete details of the search strategy have been presented in detail in Supplementary Table S1.

### Data extraction

A standardized data extraction process was applied. Two researchers independently extracted data based on the pre-designed PICOS framework table. Disputes arising during the extraction process were resolved through consultation or third-party arbitration. For data with opaque data or lacking relevant information, such as the mean, standard deviation, or standard error of the mean as described in the study, the missing data (including the sample size) were requested from the authors via email. Additionally, data were extracted from figures using WebPlotDigitizer (https://apps.automeris.io/wpd/) to extract data from studies where digital data were not available. Finally, we excluded studies from the meta-analysis that did not provide sample sizes even after contacting the authors. All steps followed PRISMA guidelines, with 10% of extracted data cross-checked for quality assurance.

### Quality and risk of bias assessments

This study employed the SYRCLE animal experiment bias risk assessment tool.^9^ Two trained researchers independently evaluated the 10 key methodological areas included in the study, including random sequence generation, allocation concealment, and blinding. Each field is judged according to the three-level standards of low risk, uncertain risk, or high risk. Disagreements in the assessment shall be resolved through consultation or third-party arbitration, with final results recorded using a dual-entry system.

### Statistical analysis

Data analysis was conducted using Review Manager software (Version 5.4.1) for statistical analyses and figure generation. Meta-analysis was conducted only when ≥3 studies were available. In the overall effect test, *P*<.05 was set as statistically significant. To account for methodological heterogeneity (e.g., differences in outcome assessment or interventions), a random-effects model was used for analysis. Since different measurement scales were used in each study to evaluate the same outcome measure, continuous data were summarized as standardized mean differences (SMD) with 95% confidence intervals (CI). Heterogeneity was assessed using the *I*^2^ statistic.^10^

## Results

### Search results

After screening 677 records (PubMed: 108, Embase: 219, Web of Science: 350; Cochrane Library and ClinicalTrials.gov: 0 each), 192 duplicate records were removed (Embase: 64, Web of Science: 128). Subsequently, 485 records were screened, of which 398 were excluded for failing to meet eligibility criteria. Ultimately, 87 full-text reports were retrieved for full-text assessment, of which 23 were unavailable. The remaining 64 reports underwent eligibility evaluation. Qualitative synthesis included 64 studies, comprising 39 stem cell-related studies (23 unmodified and 16 modified stem cells) and 25 studies on stem cell-derived exosomes/vesicles (16 unmodified exosomes/vesicles and 9 modified exosomes/vesicles) (Figure 1).

### General characteristics of the included studies

The 64 eligible studies were published between 2012 and January 13, 2025. Approximately 53% (*n* = 34) were published in 2020 or later, indicating increasing research interest in stem cell-based therapies for HIRI. These studies originated from 11 countries, with China contributing the majority (72%, *n* = 46), followed by Japan, Iran, and the Netherlands (Table 1).

**Table 1.** Characteristics of included studies and rodent HIRI models.

Author	Country	Strain (number)	block liver’s blood supply	HIRI Duration	SC Types (modification)	SC Administration Route	SC Dosage	SC therapy time	Positive surface markers
**Stem Cells**									
**Xu C et al. 2024^11^**	China	C57BL/6 mice(10)	70%	I90minR6h	hMSCs	the portal vein	1.0 × 10^5^cells/mice	after 90 min of ischemia and 6 h of reperfusion	CD90, CD73 and CD105
**Shang LC et al. 2023^12^**	China	C57BL/6 mice(104)	70%	I60minR6h	MSCs	the caudal vein	1.0 × 10^6^	30 min prior to hepatic warm I/R	NE
**Kartal B et al. 2023^13^**	Turkey	Wistar albino rat(30)	70%	I40minRx	ADSCs	liver parenchyma	1.0 × 10^6^cells/kg	following 20 min of ischemia	NE
**Chen K et al. 2022^14^**	Japan	Wistar rats(37)	70%	I60minR3/24h	ASCL	intrasplenic	1.0 × 10^6^	24 h before HIRI	CD44 and CD90
**Khosravi-Farsani S et al. 2022^15^**	Iran	BALB/c	the portal vein, hepatic artery, and bile duct of the liver	I60minR1/5h, 7d	AMSCs/BMSCs	the tail vein	1.0x10^6^	Immediately after reperfusion onset	CD29, CD73, CD105, and CD90
**Piao C et al. 2021^16^**	China	SD rats(30)	70%	I30minR24h	ADSCs	the tail vein	2.0 × 10^6^	2 h before surgery	NE
**Zheng J et al. 2020^6^**	China	C57BL/6 mice(10)	70%	I60minR6/24h	UC-MSCs	peripheral vein	1.0x10^6^/100 μl	after reperfusion	NE
**Li C et al. 2019^17^**	China	Gene editing mice	NE	I90minR6h	MSCs	the tail vein	1 × 10^6^cells/mouse	24 hours before ischemia	NE
**Zare MA et al. 2019^18^**	Iran	BALB/c(24)	70%	I60minR6/24h	BM-MSCs	the portal vein	1.0x10^6^	Immediately after reperfusion	Sca-1, CD44
**Wang X et al. 2018^19^**	China	Wistar rats(30)	the portal triad for the left and median liver lobes	6h (I60minR6h)	BM-MSCs	penis dorsal vein	1.0x10^6^	30 min prior to hepatic warm I/R	CD29, CD44 and CD90
**Qi X et al. 2018^20^**	China	C57/B6 and inbred Buffalo rats	the right hepatic pedicle	I60minR6h, 1/3/5d	hiPSC-MSCs	the portal vein	5 × 10^4^ cells/100µL PBS	after reperfusion	NE
**Li S et al. 2018^21^**	China	SD rats	the hepatic artery and portal vein to the left lateral and median lobes	I60minR12/24/72h, 14d	MSCs	the portal vein	3.0 × 10^5^	Reperfusion was initiated	CD29, CD44, and CD90
**Isbambetov A et al. 2016^22^**	Japan	F344 wild-type rats(60)	70%	I60minR1/3/7d	BM-MSCs	the tail vein	1.5x10^6^	15 min after initiating reperfusion	NE
**Saat T C et al. 2016^23^**	Netherlands	C57BL/6(49)	70%	I60minR6/48h, 5d	MSC	the tail vein	2.0 × 10^5^	2 hours before and 1 hour after ischemia	NE
**Wang X et al. 2016^24^**	China	SD rat(60)	NE	I40minR1/2/3W	hAD-MSCs	the tail vein	0.5 mL	2 weeks after HIRI model	NE
**Nowacki M et al. 2015^25^**	Poland	Wistar rats(20)	the portal triad under the left lateral lobe and left median lobe	I60minR3M	BM-MSCs	intraportally	1.0x10^6^	After IRI	NE
**Lee SC et al. 2015^26^**	Korea	BALB/c mice(100)	the portal vein, hepatic artery, and bile duct above the branching to right lateral lobe.	I45minR3/6/12/24h	ASCs	the tail vein	1.0x10^6^	within 1 hour of HIRI	CD90
**Fouraschen SM et al. 2015^27^**	Netherlands	C57BL/6 mice(32 + 16)	70%	I90minR6/24h, I60minR48h	MSC-CM	intraperitoneally	200 μL	at the end of surgical procedure.	NE
**Saidi R F et al. 2014^28^**	USA	C57BL/6(28)	70%	I60minR6/12/18/24h	HADMSCs	the tail vein	1–2 million	30 minutes before ischemia	CD105, CD29, CD44, CD90
**Saito Y et al. 2013^29^**	Japan	BALB/c nu-nu mice	the hepatoduodenal ligament	I15minR6/24h, I20minR24h	h-ADSCs	the tail vein	1.0x10^5^/0.1 ml/mouse	after the hepatectomy	CD29, CD44, CD73, CD90, CD105, and CD166
**Sun CK et al. 2012^7^**	Taiwan, China	Fisher rats(30)	the left lobe liver	I60minR72h	ADMSCs	venous	1.2x10^6^	immediately, 6 hrs, and 24 hrs after reperfusion	CD-90 and CD-29
**Seki T et al. 2012^30^**	Japan	Wistar rats	the hepatoduodenal ligament	I15minR1/2d	ADSCs	the penile vein	2x10^6^cells/rat	After releasing the clamp	NE
**Pan GZ et al. 2012^31^**	China	SD rats	the hepatic artery, portal vein, and bile duct to left lateral and median lobes of liver.	I60minR6h, 1/2/3/5d	MSCs	the tail vein	3.0x10^6^	2 h before the surgical operation	CD54 and CD90
**Stem Cells Modification**									
**Chen H et al. 2024^32^**	China	C57BL/6 mice	the artery/portal vein blood supply to left and middle liver lobes.	I90minR2/6/12/24/48h	UC-MSCs(MMCLs)	the tail vein	NE	after the initiation of reperfusion	NE
**Sheng MW et al. 2024^33^**	China	C57 mice(24)	70%	I90minR6h	MSCs(CD47-overexpressed)	the tail vein	1.0x10^6^	24 h before surgery	NE
**Ko SF et al. 2023^34^**	Taiwan, China	SD rats (50)	NE	I60minR18/72h	tacrolimus-ADMSCs	intravenous	1.2 × 10^6^	3h after IR	NE
**Zhang Q et al. 2023^35^**	China	C57BL/6J(27)	70%	I60minR6h/7d	MenSCs(Interferon-γ)	intravenous	1.0x10^6^/100 μL	1h before the surgery	CD29, CD73, CD90, and CD105
**Tian X et al. 2023^36^**	China	SPF SD rats(36)	70%	I80minR24h	BMMSCs(HO-1)	caudal and portal vein	2.0 × 10^6^	1 day before surgery and during reperfusion	CD90, CD29, and RT1A
**Owen A et al. 2022^37^**	UK	C57BL/6 mice	NE	I60minR24h	PDGFRα/Sca-1 (PaS) sorted MSC	intraperitoneal	1.0 × 10^6^	One hour prior to the induction of ischemia	NE
**Lin Y et al. 2022^38^**	China	Wistar albino rats	NE	I45minR24h	BMSCs(Baicalin Combined)	the portal vein	1.0x10^6^	after reperfusion	CD29, CD44 and CD105
**Sahu A et al. 2021^39^**	Korea	wild-type ICR mice(25)	70%	I90minR24h	MSC(Nanozyme Impregnated)	hepatic vein	1 × 10^5^cells/mice	90 min post hepatic ischemia	CD90 and CD29
**Li Q et al. 2021^40^**	China	Wistar rats(80)	70%	I60minRx	SOD2-BMMSCs	the tail vein	1 × 10^6^PKH26-labeled	NE	Bax, Bcl-2 and caspase-3
**Tan Y et al. 2021^41^**	China	SPF SD rats	the left outer and left middle hepatic artery trunks	I30minRx	BMSCs(TNF-α)	the portal vein	0.5ml	opened the blood vessels	CD79, CD45, CD90, and CD29
**Zheng J et al. 2019^42^**	China	C57BL/6 mice (36-42)	70%	I90minR24h	UC‐MSCs(rapamycin)	the peripheral vein	1 × 10^6^cells/100 µL per mouse	After model was established	CD105, CD44, CD29, CD90, CD73 and CD166
**Liu J et al. 2019^43^**	China	SD rats(90)	70%	I60minR12h	ADMSCs(Mild hypothermia combined)	the femoral vein	1.0x10^9^	30min before ischemia	NE
**Sun Y et al. 2018^44^**	China	SD rats(24)	70%	I90minR6/24/48h	UC-MSCs(Spheroid-cultured)	intraperitoneal	3 × 10^6^ per rat	after reperfusion	NE
**Feng J et al. 2018^45^**	China	SD rats(44)	70%	I60minR6/24h	hBM-MSCs(Intravenous Anesthetics)	the portal vein	5.0 × 10^4^	NE	NE
**Qiao PF et al. 2015^46^**	China	SD rats(32)	70%	I60minR24h	MSC/HSP-MSCs(HSP)	the portal veins	1.0x10^6^	The clamp was removed	NE
**Fu J et al. 2014^47^**	China	SD rats(30)	the portal triad	I30minR6/24h	MSCs(N-acetyltransferase 8)	the tail vein	3.0x10^6^	reperfusion was initiated	CD29, CD44, CD73, CD90, and CD105
**Exosomes/Vesicles**									
**Li H et al. 2024^48^**	China	Kunming mice(30)	the mid and left lobes’ portal vein and hepatic artery.	I60minR6h	BMSC-Evs	the tail vein	50 μg in 100 μL PBS	24 hours before surgery	CD73, CD90, CD63, TSG101 and CD9,
**Li H et al. 2023^49^**	China	KM mice(30)	the portal vein and hepatic artery of the middle and left lobes.	I60minR3/6/9h	BMSC-exosomes miR-25-3p	the tail vein	50 μg in 100 μL PBS	24 h before surgery	CD73, CD90, CD63, TSG101 and CD9
**Gong Y et al. 2023^50^**	China	SD rats(30)	70%	I60minR24h	ADSCs-exo	the tail vein	30mg/kg	NE	CD90, CD105, CD29, and CD73
**Zhang Y et al. 2022^51^**	China	SD rats (n=41)	70%	I60minR2/6h	ADSCs-exo	the portal vein	50 µl (30 µg)	NE	CD73, CD90 and CD105
**Piao C et al. 2022^52^**	China	SD rats(24)	the median and left lateral liver lobes	IxR24h	ADSCs-Exo	the tail vein	2 × 106 ADSCs or 100 gADSCs-Exo	immediately after the operation	NE
**Zhang Q et al. 2021^53^**	China	SD rats(24)	70%	I30minR24h	ADSCs-exo	the tail vein	100 μg	After surgery	CD29, CD44 and CD90
**Calleri A et al. 2021^54^**	Italy	C57BL/6 mice(38)	70%	I90minR6h	HLSC-EV	the tail vein	3 × 10^9^ or 7.5 × 10^9^	Immediately after reperfusion	CD9, CD63, CD81, CD29, CD44, CD105 and CD49e, CD142, CD146, SSEA-4, and MCSP
**Zheng J et al. 2020^55^**	China	C57BL/6, B6 mice	70%	I90minR6h	UC-MSC-EVs	peripheral intravenous	1.0x10^6^	after reperfusion	CD63, CD9 TSG101, and ALIX
**Xie K et al. 2019^56^**	China	C57BL/6 mice(36)	the artery/portal vein blood supply to the left and middle liver lobes.	I90minR6/12/24h	hUCB-MSCs-exo	intravenously	10 μg	following 90 min of ischemia	NE
**Xie K et al. 2019^57^**	China	C57BL/6 mice(42)	the artery/portal vein blood supply to the left and middle liver lobes.	I90minR6/12/24h	hUCB-MSCs-exo	the portal vein	2.5 × 10^12^ particles for each moue	after the initiation of reperfusion	CD29, CD105, CD9 and CD63
**Nong K et al. 2019^58^**	China	SPF SD rats(43)	the medial and left hepatic hila	I45minR12h	MSC-exo	the tail vein	500 μg of exosomes dissolved in 1 mL of PBS solution	24 hours before the surgery.	CD29 and CD90
**Yao J et al. 2019^59^**	China	SD rats	70%	I90minR6/24h	hucMSC-EVs	the tail vein	10 mg/kg	immediately after reperfu sion	CD29, CD90, CD105, CD73, CD63 and CD9
**Anger F et al. 2019^60^**	Germany	C57BL/6 mice(25)	70%	I90minR1/2/3d	hMSCs-EV	V. cava inferior	NE	After a midline laparotomy	CD105, CD90, CD73, CD9 and CD63
**Du Y et al. 2017^61^**	China	C57Bl/6 mice(18)	70%	I60minR1/3/6/12/24h	hiPSC-MSCs-Exo	inferior vena cava	2.5 × 10^12^ particles	immediately after the initiation of reperfusion	CD29, CD73, CD90 and CD105
**Nong K et al. 2017^62^**	China	SD rats	70%	I60minR1/3/6/12/24h	hiPSCMSCs-Exo	inferior vena cava	600 μg	immediately after the initiation of reperfusion	CD29, CD73 and CD90
**Haga H et al. 2017^63^**	USA	C57BL/6(25)	70%	I90minR1/3/6h	MSC-EV	the tail vein	2.0x10^10^	30 minutes prior to ischemia	NE
**Exosomes/Vesicles Modification**									
**Zhang B et al. 2024^64^**	China	C57BL/6 mice	70%	I90minR1/3/6h	Baicalin-pretreated BMSCs-Exos(Ba-Exo)	the tail vein	NE	after 90 min of ischemia and 6 h of reperfusion	CD9, CD63, CD81, TSG101
**Piao C et al. 2024^65^**	China	SD rats(30)	the Glisson’s blood supply to the middle and left lobes of the liver.	I30minRx	Metformin and ADSCs-Exo complex (Met-Exo)	the tail vein	100ug	immediately after the operation	ALIX, CD9, CD81 and TSG101
**Li R et al.2024^66^**	China	C57BL/6 albino mice	70%	I60minR1/3/7/14d	Phosphatidylserine-mediated MSC-Evs	the tail vein	100 µg/100 µL per mouse	after 12 h of reperfusion	CD9, TSG101, Alix, CD73, CD90 and CD105
**Miao L et al. 2024^67^**	China	C57BL/6(25)	70%	I60minR6/24h	MSC-EVs containing GAS6	the tail vein	2 × 10^10^ particles/body	immediately after the restoration of blood supply	Alix, TSG101, CD9
**Sameri MJ et al. 2022^68^**	Iran	BALB/C mice(24)	70%	I60minR3h	MSC-Exo/H2S-Exo(NaHS (1 mmol))	the tail vein	100 mg	ending the time of ischemia	CD9 and CD63
**Li X et al. 2022^69^**	China	SD rats(30)	70%	I80minR24h	BMMSCs-exo(Heme Oxygenase-1)	tail and portal vein	2.0 × 10^6^	1 day before surgery and immediately after reperfusion	CD29, CD90, and RT1A
**Zhang L et al.2020^70^**	China	SD rats(48)	75%	I60minR24h	UC-MSCs(MiR-20a-containing exosomes)	NE	20 mg	NE	CD73, CD90, CD105, CD34 and CD45
**Yang B et al. 2020^71^**	China	C57BL/6 mice	70%	I60minR6h	BMMSC-Derived Hepatocyte-Like Cell Exosomes(MSC-Heps-Exo)	the tail vein	100 μg	before and after the operation	CD29, CD44, CD105, Sca-1, CD9, CD63, and TSG101
**Wei X et al. 2020^72^**	China	SD rats(40)	the portal venous and arterial blood supply to central and left liver lobes.	I90minR6h	MSC-exo(glycyrrhetinic acid(GA))	intraperitoneal shot	NE	NE	NE

HIRI, Hepatic ischemia-reperfusion injury; SC, stem cells; SD, Spraque–Dawley; I, ischemia; R, reperfusion; HSP, Heat shock pretreatment; NE, not evaluated; X, not explicitly stated.

### Characteristics of HIRI models

#### Animal species

All studies employed rodents, including mice (24 studies, 37.5%) and rats (40 studies, 62.5%), with males constituting at least 82.8% (53/64) of subjects. The C57BL/6 strain (17 studies) was predominant in mouse models due to its stable genetic background and suitability for metabolic studies. In contrast, rat studies primarily used Sprague-Dawley (SD, 23 studies) and Wistar (7 studies) strains, favored for their larger organ size and surgical feasibility.

#### Disease models

The animal models focused on HIRI (64 studies), with the following distribution: Isolated HIRI: 81.3% (52/64), HIRI + partial hepatectomy (PH): 12.5% (8/64), HIRI + PH + orthotopic liver transplantation: 1.6% (1/64), HIRI + hypoxia/reoxygenation (H/R): 4.7% (3/64). Among HIRI models, 51.6% (33/64) adopted the 70% liver ischemia standard.

#### HIRI models

There were significant differences in ischemia and reperfusion times in the model (Table 1). The ischemic time was most common at 60 minutes (48.4%,31/64), followed by 90 minutes (28.1%,18/64), ranging from 15 to 90 minutes. The reperfusion time was mainly 6 hours (40.6%,26/64) and 24 hours (51.6%,33/64), and in some studies, it was extended to 48 hours, 72 hours, or even 3 months. Chinese studies mostly adopt 60-minute ischemia + 6/24 hour reperfusion, while Japanese studies tend to favor short-term ischemia (15-45 minutes) and long-term reperfusion (1 day to several days).

### Intervention characteristics

#### Type and source of stem cells

This study systematically analyzed the characteristics of stem cells type and their sources used for treating HIRI (Table 1). The results demonstrated that MSCs served as the primary therapeutic option (96.9%), with bone marrow-derived (BM-MSCs), adipose-derived (ADSCs), and umbilical cord-derived MSCs (UC-MSCs) being the most prevalent subtypes. Notably, approximately 25% (16/64) of the studies employed genetically modified or pharmacologically combined engineered stem cells to enhance therapeutic efficacy. Regarding cells origin, human-derived stem cells predominated in clinical applications, while rodent-derived cells were primarily utilized in preclinical investigations.

#### Stem cells and exosomes/vesicles modification

This meta-analysis evaluated 16 studies involving stem cells modification and 9 involving stem cell-derived exosome/vesicle modification for the treatment of HIRI. Current modification strategies can be broadly classified into four categories: (1) genetic engineering, including viral vector-mediated overexpression of protective genes (e.g., CD47, HO-1),^33^^,^^36^^,^^40^^,^^47^^,^^67^^,^^69^ miRNA modulation through gene transfection,^70^ and surface marker-based selection to enrich specific MSC subpopulations;^37^ (2) pharmacological combination therapies, involving immunosuppressants,^34^^,^^42^ active components of traditional Chinese medicine,^38^^,^^64^^,^^72^ anesthetics,^45^ cytokines,^35^^,^^41^ and metabolic regulators^65^ (3) physicochemical preconditioning, such as hypothermic culture,^43^ spheroid culture,^44^ heat shock,^46^ phosphatidylserine-mediated membrane modification,^66^ and hepatocyte-like cell-derived exosomes;^71^ and (4) nanomaterial com­posites, including nanozyme loading,^39^ biomembrane-nanocarrier hybrid systems,^32^ and exosome-drug complexes.^68^ These modifi­cations enhanced targeting specificity, anti-inflammatory capacity, and cell survival. For instance, CD47 overexpression inhibiting pyroptosis and HO-1 overexpression enhancing anti-inflammatory efficacy.

### Stem cells administration: route, dosage, and timing

#### Administration routes

The included studies employed diverse stem cells delivery methods, primarily intravenous (66%, 42/64) and intraperitoneal routes (Table 1). Intravenous administration included the tail vein (33 studies), peripheral veins,^6^^,^^42^^,^^55^ penile vein,^19^^,^^30^ inferior vena cava,^60–62^ and femoral vein,^43^ facilitating systemic distribution. Hepatic-targeted delivery (22%, 14/64) was achieved via portal vein (13 studies) and hepatic vein^39^ injections. Intraperitoneal administration^27^^,^^37^^,^^44^^,^^72^ was mainly used for modified stem cells (e.g., PDGFRα/Sca-1-sorted MSCs, spheroid-cultured UC-MSCs, glycyrrhetinic acid (GA)-modified MSC-exosomes), whereas localized injections (intrasplenic^14^ and intrahepatic^13^) were applied for adipose-derived stem cells (ADSCs). Notably, genetically modified stem cells (e.g., CD47-overexpressing MSCs, baicalin-combined BMSCs) predominantly utilized intravenous routes, whereas exosome-based therapies mainly administered tail vein delivery (64%, 16/25).

#### Stem cells dose

Stem cells dosage varied depending on cell type, animal model and modification status (Table 1): Unmodified stem cells: The typical dosage is 1 × 10^5^ to 3 × 10^6^ cells/animals, most commonly 1 × 10^6^ cells/mouse; Some studies used higher doses (e.g., 1 × 10^9^ cells for cold-treated ADSCs). Modified stem cells: The dose of engineered MSCs (e.g., SOD2-BMMSCs, TNF-α-pretreated BMSCs) is mostly 1–2 × 10^6^ cells, whereas exosome therapy required lower doses (such as 30–100 μg protein or 10^10^–10^12^ particles). Exosome/vesicle therapy: The dosage is usually standardized according to the protein content (such as 50 μg BMSC-EVs) or the number of particles. Modified exosomes (such as EVs carrying GAS6) may require a higher concentration (2 × 10^10^ particles).

#### Administration time

Interventions were classified based on timing relative to HIRI: Pretreatment (before ischemia, 25%, 16/64 studies), using strategies such as mild hypothermia combined ADMSCs, CD47-overexpressed MSCs, HO-1 BMMSCs. Interferon-γ (MenSCs) to enhance homing ability; Perioperative period (during HIRI): 22% (14/64) of the studies administered drugs (such as BM-MSCs, ADSCs) at the beginning of reperfusion to exert immediate anti-inflammatory effects; Delayed intervention (after HIRI): 42% (27/64) of the studies administered the drug several hours to several weeks after injury, with a focus on liver tissue regeneration and repair. Modified stem cells were often used in delayed regimens, whereas exosome therapies were predominantly administered perioperatively.

#### Stem cells surface markers

Stem cells express characteristic surface markers: the core positive markers are CD90, CD73 and CD105 constitute the core triad markers of mesenchymal stem cells; Genetically modified stem cells retain these core markers while exhibiting modification-specific markers (e.g., CD29/CD90 in HO-1-BMMSCs).

### Outcome index

#### Liver function

MSCs markedly improved hepatic function following HIRI, as evidenced by reductions in serum biochemical markers (ALT, AST, LDH, and ALP). Across 64 included studies, 81% (52/64) demonstrated that MSCs and their exosomes effectively suppressed HIRI-induced elevation in liver enzymes and preserved hepatocyte functional homeostasis **(Supplementary Table S2)**, highlighting their robust hepatoprotective effects.

#### Pathological features

Histopathological analyses consistently showed that MSCs alleviated HIRI-induced hepatic tissue injury. Zhang et al.^53^ reported improvements in ultrastructural hepatocyte injuries (e.g., nuclear condensation) and increased mitochondrial biogenesis. Other studies^6^^,^^35^^,^^69^ observed decreased Suzuki’s score. While kartal et al.^13^^,^^21^ noted attenuation of portal inflammation, hepatocyte edema, cytoplasmic deformation and coagulative necrosis. Additionally, Zare et al.^18^^,^^26^^,^^28^^,^^33^^,^^35^^,^^38^^,^^54^^,^^55^^,^^61^^,^^62^^,^^68^^,^^71^ reported reduced hepatocyte congestion, hepatocyte vacuolation, necrosis and inflammatory infiltration. Ultimately, multiple studies^48^^,^^49^ have shown that MSCs can significantly reduce the area of necrotic regions and maintain structural integrity; It simultaneously inhibits end-stage lesions such as coagulative necrosis^13^ and fibrosis.^24^^,^^58^

#### Oxidative stress and inflammation regulation

Meta-analysis of the included studies revealed that MSCs exert protective effects through the following key mechanisms: (1) Attenuation of oxidative stress^7^^,^^16^^,^^38–42^^,^^53^^,^^59^^,^^62^^,^^63^^,^^65^ through ROS scavenging,^14^^,^^71^ inhibition of mitochondrial fission,^12^ restoration of mitochondrial function,^32^ upregulation of PINK1-mediated mitophagy,^6^ and reduction of lipid peroxidation;^51^ (2) Modulation of inflammatory responses^7^^,^^16^^,^^20^^,^^34^^,^^52^^,^^58^^,^^67^ through downregulation of pro-inflammatory cytokines (TNF-α, IL-6, IL-1β),^17^^,^^23^^,^^26^^,^^36^^,^^37^^,^^44^^,^^54^^,^^55^^,^^66^^,^^69^^,^^72^ promotion of anti-inflammatory IL-10 secretion,^37^ reduction of neutrophil infiltration,^21^^,^^26^^,^^59^ regulation of Treg/Th17 balance,^56^^,^^64^ and reprogramming of intrahepatic transcriptional profiles of inflammation-related genes post-IRI,^60^ including downregulation of inflammatory genes (e.g., TNFA and IL1RN^27^)

#### Cell apoptosis and regeneration

MSCs improve liver ischemia-reperfusion injury through a dual mechanism: (1) Anti-apoptotic, as they significantly suppresses apoptosis^6^^,^^7^^,^^11^^,^^18^^,^^20^^,^^32–36^^,^^38–40^^,^^44^^,^^48^^,^^49^^,^^53^^,^^55^^,^^57^^,^^58^^,^^62^^,^^63^^,^^66–71^ by down-regulating pro-apoptotic proteins(Caspase-3 and BAX), upregulating anti-apoptotic protein Bcl-2 and reduce TUNEL-positive cells; and (2) Pro-regenerative, as they promotes hepatocyte regeneration^20^^,^^22^^,^^27^^,^^29^^,^^31^^,^^47^^,^^50^^,^^52^^,^^60^^,^^61^ by increasing PCNA-positive cells,^28^ elevating mitotic index^30^ and enhancing hepatocyte growth factor (HGF) expression.^24^ Additionally, inhibition of α-SMA expression^24^ mitigates progression toward liver fibrosis.

### SYRCLE’s risk of bias tool

**Figure 2.**
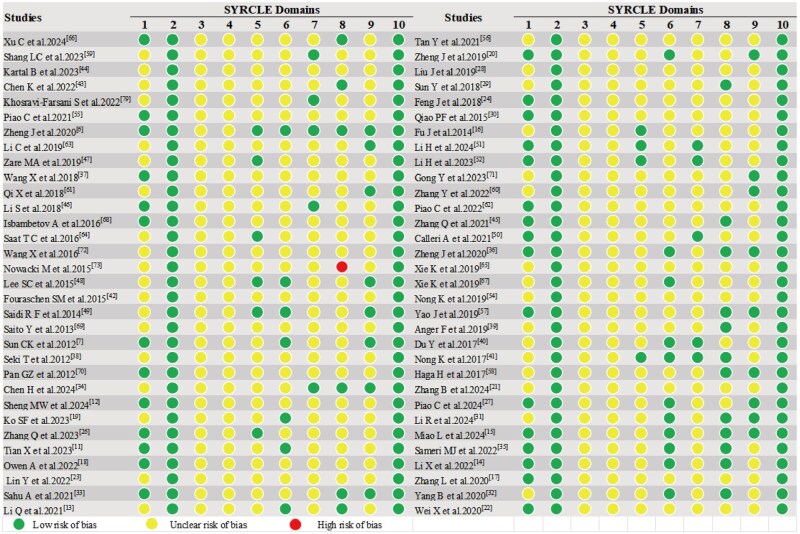
The methodological quality of each animal study was examined through SYRCLE’s RoB tool.

The SYRCLE-based risk of bias assessment for all 64 included studies is summarized in Figure 2. Overall, 45.3% of studies reported random sequence generation, with four studies specifically detailing their randomization methods.^33^^,^^45^^,^^48^^,^^49^ Baseline characteristics indicated a low risk of bias. However, none of articles described randomization of animal housing or allocation concealment, raising concerns about potential selection bias. One study had incomplete outcome data.^25^No evidence of selective outcome reporting or other systematic biases was identified. Notably, most studies inadequately reported key methodological elements, particularly randomization and blinding procedures.

### Meta-analysis

A meta-analysis of 32 eligible studies (41 groups) using 70% hepatic ischemia-reperfusion injury (HIRI) models, evaluated the following outcome measures: ALT, AST, Suzuki’s score, necrotic area ratio per visual field, serum TNF-α levels, and related proteins (Bcl-2, BAX, c-Caspase 3). Studies were stratified by ischemia duration into I45min (1 group), I60min (29 groups), I80min (1 group), and I90min (10 groups). Given the sample size distribution, the analysis primarily focused on the I60min and I90min subgroups to ensure statistical power.

**Figure 3.**
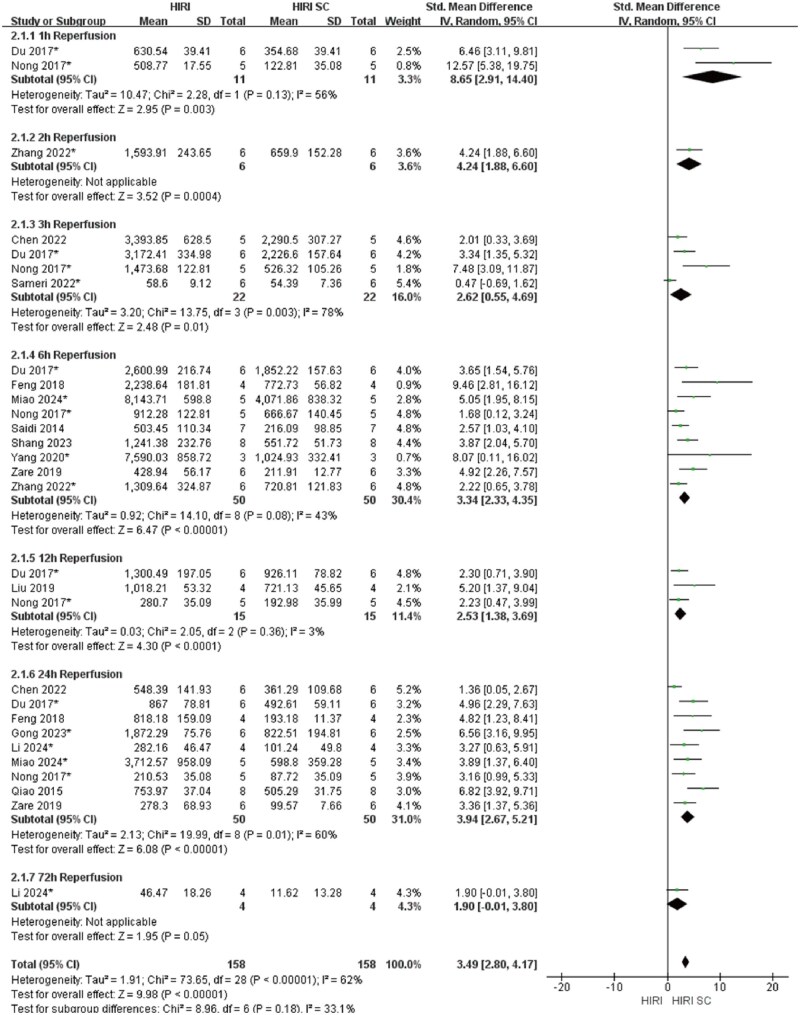
Forest plots showed that in 29 studies with 70%HIRI and 60 minutes of ischemia time, the liver function marker ALT was significantly reduced after stem cell intervention. *Stands for exosomes/vesicles or exosomes/vesicles modification.

**Figure 4.**
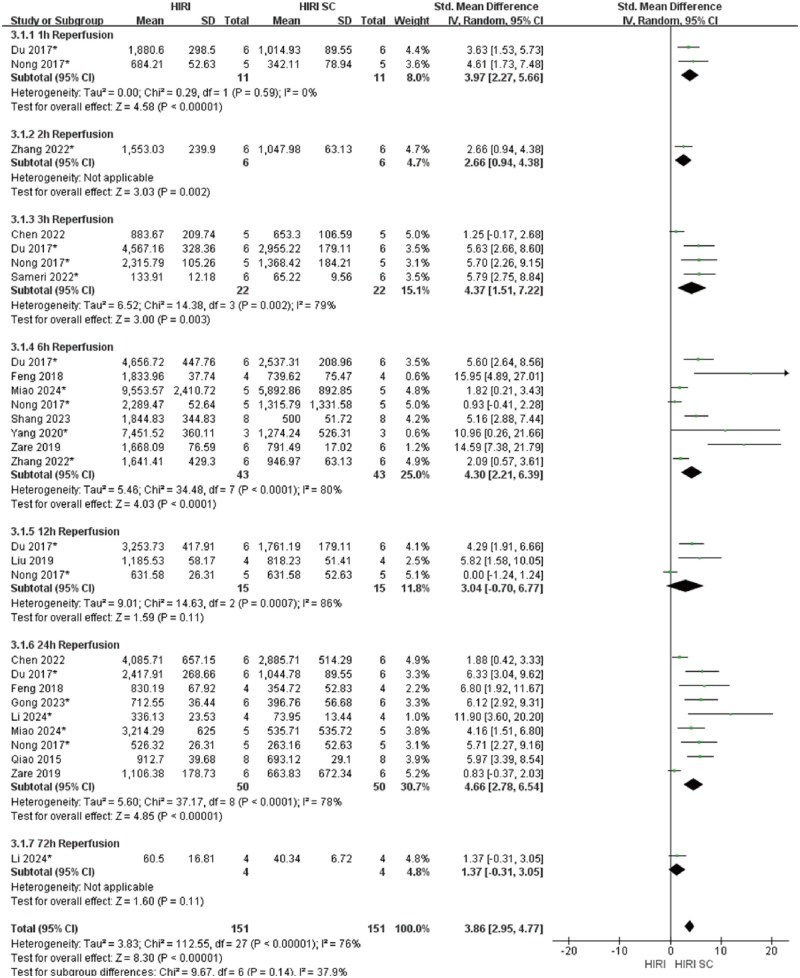
Forest plots showed that in 28 studies with 70%HIRI and 60 minutes of ischemia time, the liver function marker AST was significantly reduced after stem cell intervention. *Stands for exosomes/vesicles or exosomes/vesicles modification.

**Figure 5.**
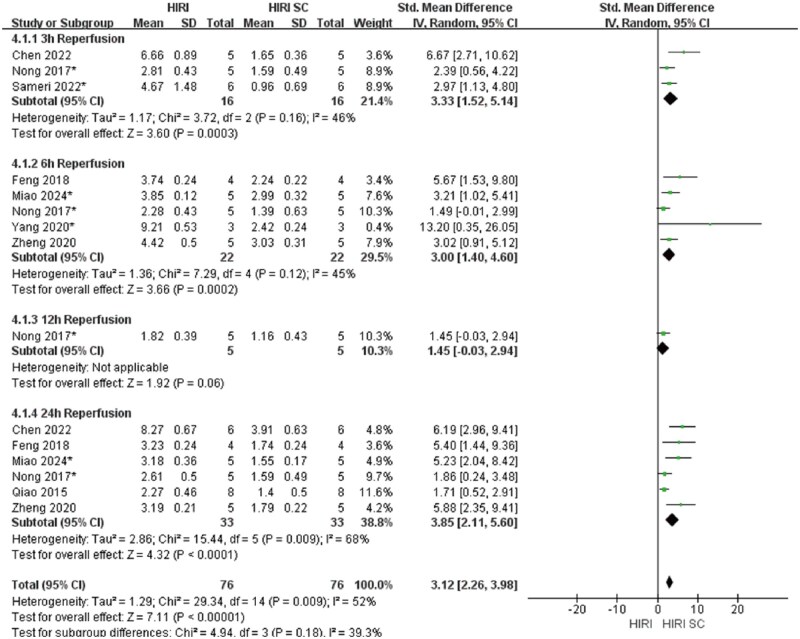
Forest plots showed that in 15 studies with 70%HIRI and 60 minutes of ischemia time, the Suzuki’s score was significantly reduced after stem cell intervention. *Stands for exosomes/vesicles or exosomes/vesicles modification.

**Figure 6.**
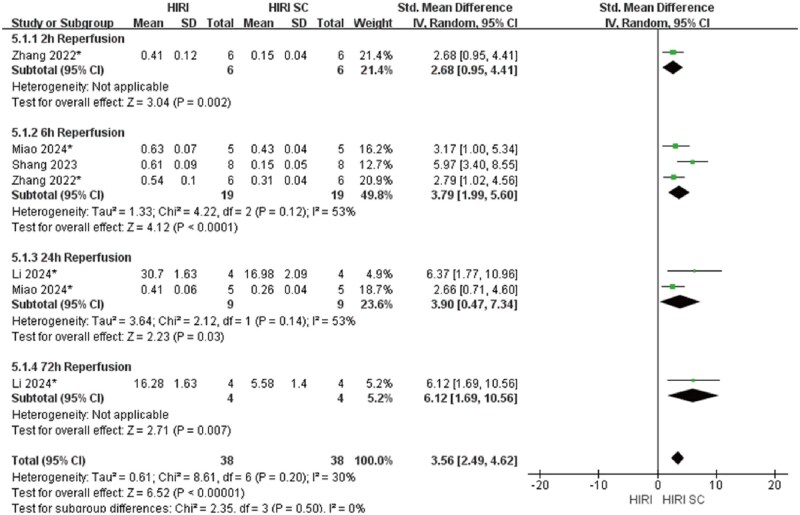
Forest plots showed that in 7 studies with 70%HIRI and 60 minutes of ischemia time, the necrotic area ratio per visual field was significantly reduced after stem cell intervention. *Stands for exosomes/vesicles or exosomes/vesicles modification.

**Figure 7.**
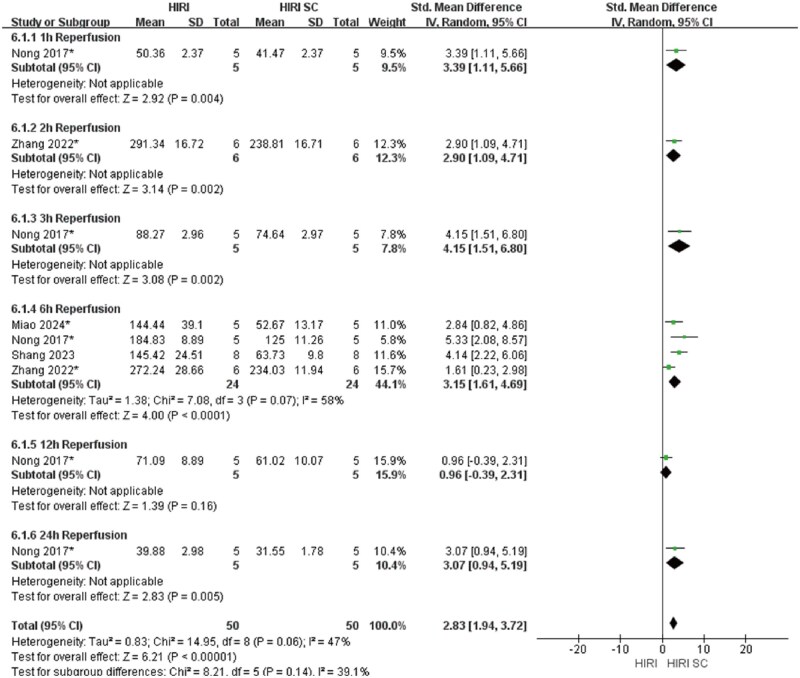
Forest plots showed that in 9 studies with 70%HIRI and 60 minutes of ischemia time, the Serum TNF-α was significantly reduced after stem cell intervention. *Stands for exosomes/vesicles or exosomes/vesicles modification.

**Figure 8.**
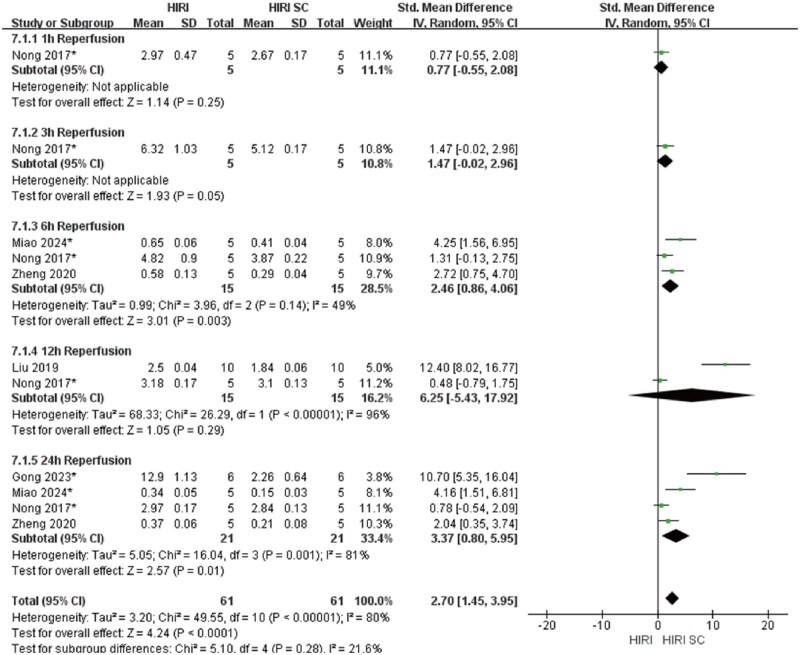
Forest plots showed that in 11 studies with 70%HIRI and 60 minutes of ischemia time, the c-Caspase 3 expression was significantly reduced after stem cell intervention. *Stands for exosomes/vesicles or exosomes/vesicles modification.

In 70%HIRI rodent models with 60-minute ischemia, MSCs significantly reducted ALT and AST compared with controls (ALT: SMD = 3.49, 95% CI: 2.80–4.17, *P* < .00001; AST: SMD = 3.86, 95% CI: 2.95-4.77, *P*<.00001) (Figures 3 and 4). High heterogeneity indices (ALT: *I*^2^ = 62%; AST: *I*^2^ = 76%) reflected variability in stem cells sources, preparation, and administration. ALT showed marked heterogeneity at 3 h (*I*^2^ = 78%) and 24 h (*I*^2^ = 60%) reperfusion timepoints, while AST showed heterogeneity at 3 h (*I*^2^ = 79%), 6 h (*I*^2^ = 80%), 12 h (*I*^2^ = 86%), and 24 h (*I*^2^ = 78%). MSCs also reduced Suzuki’s score (SMD = 3.12, 95% CI: 2.26-3.98, *P*<.00001, *I*^2^ = 52%), necrotic area ratio (SMD = 3.56, 95% CI: 2.49-4.62, *P*<.00001), serum TNF-α (SMD = 2.83, 95% CI: 1.94-3.72, *P*<.00001), and c-Caspase 3 expression (SMD = 2.70, 95% CI: 1.45-3.95, *P*<.0001) (Figures 5–8). Funnel plots indicated no publication bias (Supplementary Figure S1).

Similarly, in 70%HIRI models with 90-minute ischemia, MSCs significantly reduced ALT (SMD = 4.09, 95% CI: 2.59–5.59, *P* < .00001, *I*^2^ = 75%) and AST (SMD = 3.78, 95% CI: 3.02–4.53, *P*<.00001, *I*^2^ = 19%) (Supplementary Figure S2). ALT showed high heterogeneity at 6 h (*I*^2^ = 81%) and 24 h (*I*^2^ = 62%). Funnel plots indicated no publication bias.

## Discussion

Hepatic ischemia-reperfusion injury (HIRI) is a common and severe complication encountered during hepatic surgeries such as hepatectomy and liver transplantation. Although timely restoration of blood flow during surgery can partially mitigate ischemic damage, the reperfusion process itself paradoxically exacerbates hepatic injury by triggering oxidative stress, inflammatory cascades, and cell death, potentially leading to irreversible massive necrosis and severely compromising postoperative recovery and long-term outcomes. Currently, no specific and effective strategies are available for the prevention or treatment of HIRI in clinical practice. In recent years, therapeutic approaches based on MSCs and their derivatives (such as extracellular vesicles and conditioned media) have shown remarkable potential in preclinical studies, not only significantly reducing serum liver enzyme levels and ameliorating histopathological injury, but also exerting hepatoprotective effects through multiple synergistic mechanisms-including modulation of inflammatory responses, inhibition of apoptosis, attenuation of oxidative stress, and promotion of tissue regeneration. With the continuous advancement of stem cells engineering techniques and pharmacological preconditioning strategies, the targeting specificity and therapeutic efficacy of MSC-based therapies have been further enhanced, providing new avenues for their clinical translation. By combining quantitative synthesis with qualitative appraisal, our study not only confirms the therapeutic benefits but also delineates key mechanistic insights and translational challenges that will shape the next stage of HIRI research.

## The efficacy of MSCs for HIRI: Insights from a meta-analysis

Our meta-analysis demonstrated that MSCs exert a significant therapeutic effect in alleviating HIRI, as evidenced by a marked reduction in serum transaminase levels (ALT and AST) and substantial improvements in histopathological injury severity. Specifically, the observed improvement in liver function indicators (60-minute group: ALT SMD = 3.49, AST SMD = 3.86) 90-minute group: ALT SMD = 4.09, AST SMD= 3.78) suggests a robust hepatoprotective effect. This finding aligns with previous studies reporting that MSCs restore liver function primarily through paracrine mechanisms that modulate the local microenvironment.^55^ Importantly, this protective efficacy was consistently observed across different ischemic durations (60 vs. 90 minutes) and reperfusion time points (3-24 hours), implying that MSCs may confer protection through multi-target and temporally sustained mechanisms rather than time-restricted actions.

Beyond biochemical indicators, histopathological and anti-apoptotic improvements further corroborate the therapeutic potential of MSCs. The significant reductions in Suzuki scores (SMD = 3.12) and necrotic area ratios (SMD = 3.56) indicate MSCs’ ability to directly mitigate parenchymal damage, while downregulation of apoptotic markers (e.g., c-Caspase 3 downregulation: SMD = 2.70) reveals their intrinsic anti-apoptotic mechanisms. Particularly noteworthy is the synergistic effect between inflammation suppression (e.g., TNF-α reduction: SMD = 2.83) and apoptosis inhibition, potentially mediated by the concurrent blockade of NF-κB^45^ and MAPK^11^ signaling pathways. This dual-pathway regulation underscores MSCs’ capacity to orchestrate a coordinated cytoprotective response, providing a mechanistic basis for designing future combination therapies targeting both inflammatory and apoptotic cascades in HIRI.

These findings provide pivotal insights with far-reaching implications for the field of HIRI research. While conventional strategies primarily aim to limit ischemic injury through surgical or pharmacological preconditioning, MSCs-based therapies offer a paradigm shift by actively promoting tissue repair and regeneration in addition to injury mitigation. This positions MSCs as a promising platform for precision regenerative therapy in liver surgery and transplantation. Moreover, the consistent efficacy across diverse experimental settings suggests a degree of robustness that strengthens the rationale for clinical translation.

However, the substantial heterogeneity observed across studies (*I*^2^ = 52-86%) highlights critical standardization deficiencies in current protocols. Variations in administration routes (e.g., intravenous vs. portal vein injection), dosage regimens (1 × 10^5^-1 × 10^9^ cells), discrepancies in cell sources (e.g., MSCs vs. exosomes) and preconditioning strategies (e.g., HSP-MSCs^46^) likely contribute to effect size variability. For instance, high-dose interventions (1 × 10^9^ ADMSCs^43^ or 3 × 10^9^ HLSC-EVs^54^) appear to enhance efficacy via tissue integrity preservation, transaminase suppression, and inflammatory cytokine modulation, while exosome-based therapies can achieve comparable therapeutic outcomes at significantly lower doses (100 μg),^53^^,^^71^ highlighting the concentration of bioactive components as a potential optimization target. Additionally, the temporal dynamics of reperfusion require careful consideration: early-phase improvements (3-6 h) may primarily reflect acute anti-inflammatory effects, whereas sustained benefits (>24 h) likely involve pro-regenerative processes, Extended follow-up periods are therefore warranted to fully assess the long-term tissue remodeling potential of MSCs-based therapies.

## Multimodal protective mechanisms of MSCs in HIRI

The pathogenesis of HIRI is a dynamic and multifactorial process. In its early stage, ischemic insult disrupts hepatocyte microenvironmental homeostasis, leading to adenosine triphosphate (ATP) depletion, ion pump dysfunction, and calcium ion (Ca^2+^) imbalance, which cause cellular swelling and structural damage.^73^ Although subsequent reperfusion restores oxygen delivery, it paradoxically exacerbates cellular injury by inducing a burst of reactive oxygen species (ROS), increasing intracellular Ca^2+^ concentrations, and triggering endoplasmic reticulum (ER) stress, which collectively drive hepatocyte apoptosis and necrosis.^74^ ER stress, primarily induced by calcium dyshomeostasis and hypoxia-impaired protein synthesis,^75^ activates the unfolded protein response (UPR) sensors PERK, IRE1, and ATF6, further perturbing cellular proteostasis and promoting autophagy-related gene expression that may aggravate liver injury.^76^ While these findings have shed light on the pathophysiological underpinnings of HIRI, the complexity and redundancy of its regulatory networks remain incompletely understood, posing a major barrier to the development of targeted therapies.

Within this intricate pathological landscape, stem cell-based therapy emerges as a promising multi-targeted strategy by simultaneously mitigating oxidative stress, attenuating inflammation, inhibiting cell death, and enhancing liver regeneration. Our meta-analysis demonstrated that MSCs can effectively suppress oxidative injury by scavenging ROS, inhibiting excessive mitochondrial fission,^12^ and up-regulating Pink1-dependent mitophagy.^6^ Furthermore, paracrine exosomes from MSCs reduce lipid peroxidation by delivering miR-29a-3p^69^ or activating the AMPK/SIRT1 signaling pathway.^65^ Beyond their antioxidative roles, MSCs also exert profound immunomodulatory effects: they inhibit neutrophil infiltration,^21^ downregulate pro-inflammatory cytokines (TNF-α, IL-1β), and enhance the secretion of anti-inflammatory IL-10,^37^ thereby breaking the self-amplifying cycle between oxidative stress and inflammation that underlies HIRI progression.

Importantly, MSCs does not merely prevent further hepatocyte loss but also actively promotes tissue repair and regeneration. MSCs markedly reduce hepatocyte apoptosis, as evidenced by a decrease in TUNEL-positive cells through modulation of the Bcl-2/Bax axis^18^ and downregulation of Caspase-3 expression^70^ (SMD = 2.70, *P*<.0001). Exosome-mediated miR-25-3p has also been shown to synergistically suppress apoptosis by targeting the p53/PTEN pathway.^49^ In parallel, MSCs stimulate hepatocyte proliferation via secretion of hepatocyte growth factor (HGF)^24^ and activation of sphingosine kinase signaling.^61^ Genetic and engineering advances have further potentiated these therapeutic effects, for example, HO-1-overexpressing bone marrow MSCs attenuate ferroptosis through the AMPK-Nrf2-FTH1 axis,^36^ while 3D-cultured umbilical cord MSCs enhance reparative efficacy by dampening inflammation-related gene expression.^44^

Collectively, these findings suggest that MSCs-based therapies act through an integrated network of antioxidative, anti-inflammatory, anti-apoptotic, and regenerative pathways to counteract the multifaceted injury mechanisms of HIRI. This multimodal mode of action underscores their potential to overcome the limitations of conventional single-target strategies, which have thus far shown limited efficacy in clinical settings. More importantly, by elucidating the diverse protective mechanisms mediated by MSCs, these studies not only provide critical mechanistic insights but also lay the groundwork for the rational design of next-generation cell-based or cell-free therapies, thereby facilitating the translation of MSCs from experimental models to clinical practice in the management of HIRI.

## Optimization of the therapeutic effect of MSCs strategies

To enhance the therapeutic efficacy of MSCs in HIRI, current research in stem cell engineering primarily focuses on improving their precision, stability, and lesion-targeting capacity, thereby laying the foundation for future clinical translation and application. Gene modification approaches exemplify this trend, for instance, bone marrow-derived MSCs (BM-MSCs) overexpressing superoxide dismutase 2 (SOD2) significantly attenuate liver injury by boosting cellular antioxidant defenses,^40^ while exosomes enriched with growth arrest-specific 6 (GAS6) promote macrophage efferocytosis via the MerTK-ERK-COX2 signaling cascade.^67^ Similarly, pharmacological pretreatment of MSCs can fine-tune their immunoregulatory properties; for example, baicalin-preconditioned MSCs enhance the Treg/Th17 balance through activation of the FGF21 axis.^64^ Beyond biological modification, bioengineering strategies such as nanoenzyme loading^39^ and phosphatidylserine (PS) surface modification^66^ have been shown to significantly increase MSC survival, homing efficiency, and therapeutic potency in ischemic liver tissue.

These engineering strategies are not merely incremental enhancements but represent a paradigm shift toward precision-tailored cell therapies for complex liver injuries. By enabling MSCs to more effectively withstand the hostile oxidative and inflammatory microenvironment of the reperfused liver, these modifications amplify their anti-oxidative, anti-inflammatory, and pro-regenerative effects in a synergistic manner. More importantly, they offer a proof-of-concept that MSCs can be rationally redesigned into “smart” therapeutic agents with disease-adaptive functions. However, to facilitate their clinical translation, future work must focus on establishing standardized modification protocols, scalable manufacturing systems, and rigorous safety assessments to prevent unforeseen risks such as oncogenic transformation or immunogenicity.

Exosomes, as the principal paracrine effectors of stem cells, have also garnered increasing interest as a promising cell-free therapeutic alternative. They not only recapitulate most of the beneficial effects of MSCs but also exhibit unique translational advantages, including lower immunogenicity, higher storage stability, and facile bioengineering potential.^77^ Meta-analysis evidence indicates that exosomes derived from adipose-derived stem cells (ADSCs) suppress hepatic inflammation via the miR-183/ALOX5 axis,^50^ PS-coated extracellular vesicles (EVs) enhance hepatocyte uptake efficiency,^66^ and hiPSC-MSC-derived exosomes stimulate liver regeneration through the sphingosine-1-phosphate pathway.^61^ Nevertheless, emerging data also highlight their inherent heterogeneity: for example, BMSC-EVs containing miR-27b-3p have been shown to induce abnormal hepatocyte proliferation through the Wnt/β-catenin pathway,^48^ raising safety concerns about unregulated miRNA cargo profiles.

Therefore, while exosome-based therapies hold tremendous translational promise, their clinical development must be accompanied by systematic efforts to standardize their production, dosing, and quality control, as well as to develop targeted delivery systems that can enhance lesion specificity while minimizing off-target risks. Equally crucial is the need for long-term safety evaluations in preclinical and clinical settings to identify potential tumorigenic or immunogenic effects. Addressing these challenges will be pivotal for transforming exosomes from an experimental tool into a reproducible, safe, and scalable therapeutic platform for HIRI.

Taken together, these optimized strategies spanning genetic engineering, pharmacological preconditioning, biomaterial modification, and exosome-based delivery represent a crucial step toward overcoming the current efficacy bottleneck in MSCs for HIRI. They not only deepen our mechanistic understanding of how stem cells interventions can be fine-tuned to meet the pathological complexity of HIRI but also lay a conceptual and technological foundation for the next generation of precision cell and cell-free therapies in liver surgery and transplantation settings.

## Limitations and future

Although the research results are encouraging, there are also certain limitations: Model heterogeneity: The ischemic time varies from 15 to 90 minutes, and the experimental animals include mice and rats; Insufficient reporting: Only approximately 45% (29/64) of the studies clearly described the randomization process (according to the SYRCLE bias assessment tool). Additionally, the heterogeneity of MSCs sources represents an important but unresolved issue. Although bone marrow-, adipose-, and umbilical cord-derived MSCs were included, uneven distribution across subtypes and inconsistencies in cell preparation, dosing, and administration made it difficult to determine whether intrinsic biological differences among MSCs sources affected therapeutic efficacy.

Future research should prioritize standardized HIRI models, harmonized MSCs preparation protocols, and head-to-head comparisons of MSCs sources to clarify source-dependent effects. Dose-response relationships, especially for exosomes, require systematic evaluation. Mechanistic studies integrating multi-omics analyses and comprehensive functional assessments beyond ALT/AST are needed. Ultimately, multi-center preclinical and clinical studies will be essential to develop standardized, safe, and clinically translatable MSCs-based therapies for HIRI.

## Conclusion

This meta-analysis synthesizes preclinical evidence on MSCs for HIRI, demonstrating its ability to significantly improve liver function parameters and histopathological outcomes through multimodal mechanisms. Engineered MSCs and exosomes offer promising avenues for enhanced therapeutic efficacy. However, inter-model heterogeneity and reporting inconsistencies necessitate resolution through standardized methodologies. Although our findings are derived from rodent studies, they provide essential groundwork for subsequent large-animal and human studies to establish clinically translatable strategies for HIRI treatment. Future research should prioritize dose optimization, mechanistic exploration, and preclinical validation to develop clinically viable cell-based strategies for HIRI management.

## Supplementary Material

szaf078_Supplementary_Data

## Data Availability

All data generated or analyzed during this study are included in this article.

## Supplementary information

Figure S1: Funnel plot of publication bias (70%HIRI and I60 min).

Figure S2: The ALT, AST (70%HIRI and I90 min) and Funnel plot of publication bias (70%HIRI and I90 min).

Table S1: Search strategy.

Table S2: Summary of the Mechanisms and Outcome Data of Stem Cell Therapy for HIRI.
