# Supplementary material for: Therapeutic potential of MSCs and their exosomes in hepatic Ischaemia-Reperfusion injury: a systematic review and meta-analysis of rodent studies
Source: Stem Cells Transl Med. 2026 Jan 26;15(2):szaf078. doi: 10.1093/stcltm/szaf078 (PMC12832943; doi:10.1093/stcltm/szaf078)
Supplement: szaf078_Supplementary_Data [file szaf078_supplementary_data.docx]

**Supplementary Information**

**Therapeutic Potential of MSCs and their exosomes in Hepatic Ischaemia Reperfusion Injury:**

**A Systematic Review and Meta-Analysis of Rodent Studies**

**Yanxi Mu1‡, Weixiong Zhu1‡, Wentao Ma1, Yu Cheng1, Bo Ren1, Yusheng Cheng1,2,3*, Wence Zhou1,2,3***

1. The Second Clinical Medical College, Lanzhou University, Lanzhou, 730000, China.

2. Department of General Surgery, Biotherapy, Lanzhou University Second Hospital, Lanzhou, 730000, China.

3. Gansu Province Precision Diagnosis and Treatment Engineering Research Center of Hepatobiliary Pancreatic Diseases, Gansu Province Key Laboratory of Environmental Oncology, Lanzhou, 730000, China.

**running head**: MSCs and Exosome Therapy in HIRI

Yanxi Mu, E-mail: muyx3220@163.com

Weixiong Zhu, E-mail: zhuwx21@lzu.edu.cn

‡ Yanxi Mu, and Weixiong Zhu contributed equally to this work.

Wentao Ma, E-mail:wtma1022@163.com

Yu Cheng, E-mail:chengyu10274@163.com

Bo Ren, E-mail: zzurenbo@126.com

*Corresponding author:

Yusheng Cheng, E-mail: chengyusheng2017@163.com

Wence Zhou, MD, PhD, Surgeon, Department of General Surgery, The Second Hospital of Lanzhou University, Lanzhou, China. E-mail: [zhouwc129@163.com](mailto:zhouwc129@163.com)

**Supplementary Figure S1** Funnel plot of publication bias (70%HIRI and I60 min, I: ischemia)

**Supplementary Figure S2** The ALT/AST and Funnel plot of publication bias (70%HIRI and I90 min)

**Supplementary Table S1** Search strategy

**Supplementary Table S2** Summary of the Mechanisms and Outcome Data of Stem Cell Therapy for HIRI

**Supplementary Figure S1**


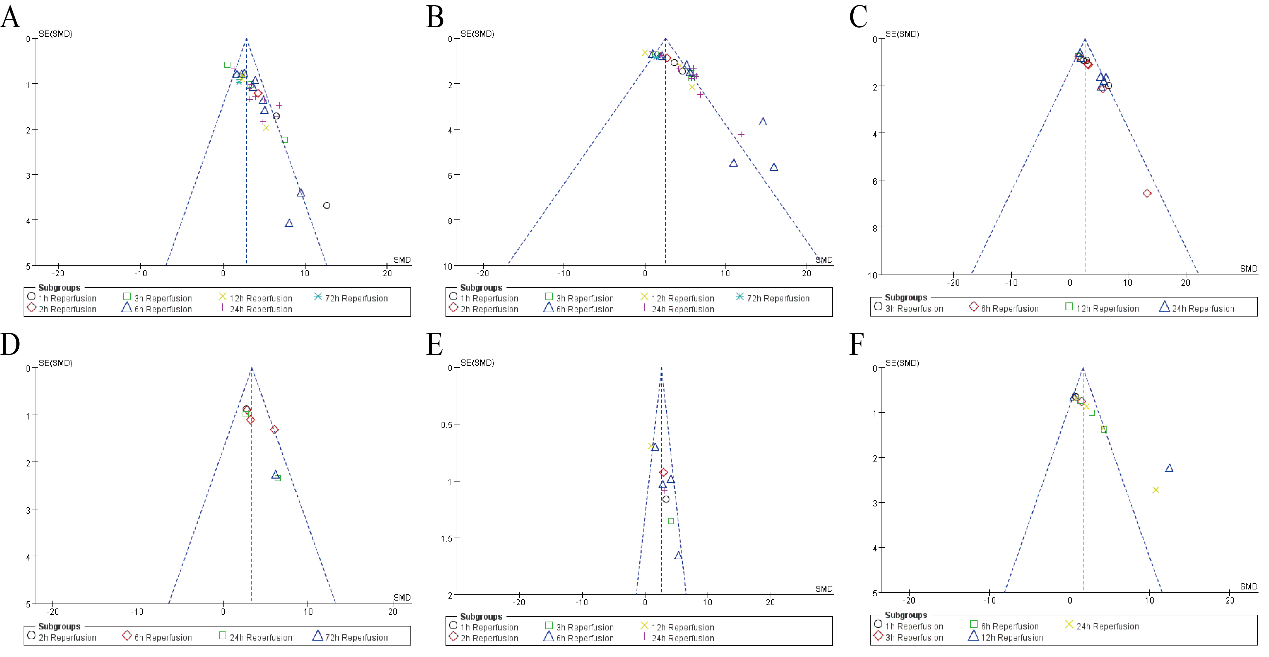


**Supplementary Figure S1.** Funnel plots assessing publication bias in studies involving 70% HIRI with 60 minutes of ischemia. The plots correspond to the meta-analyses of ALT (A), AST (B), Suzuki’s score (C), necrotic area ratio (D), serum TNF-α (E), and cleaved caspase-3 (F). The funnel plots of all six meta-analyses showed no evidence of publication bias.

**Supplementary Figure S2**


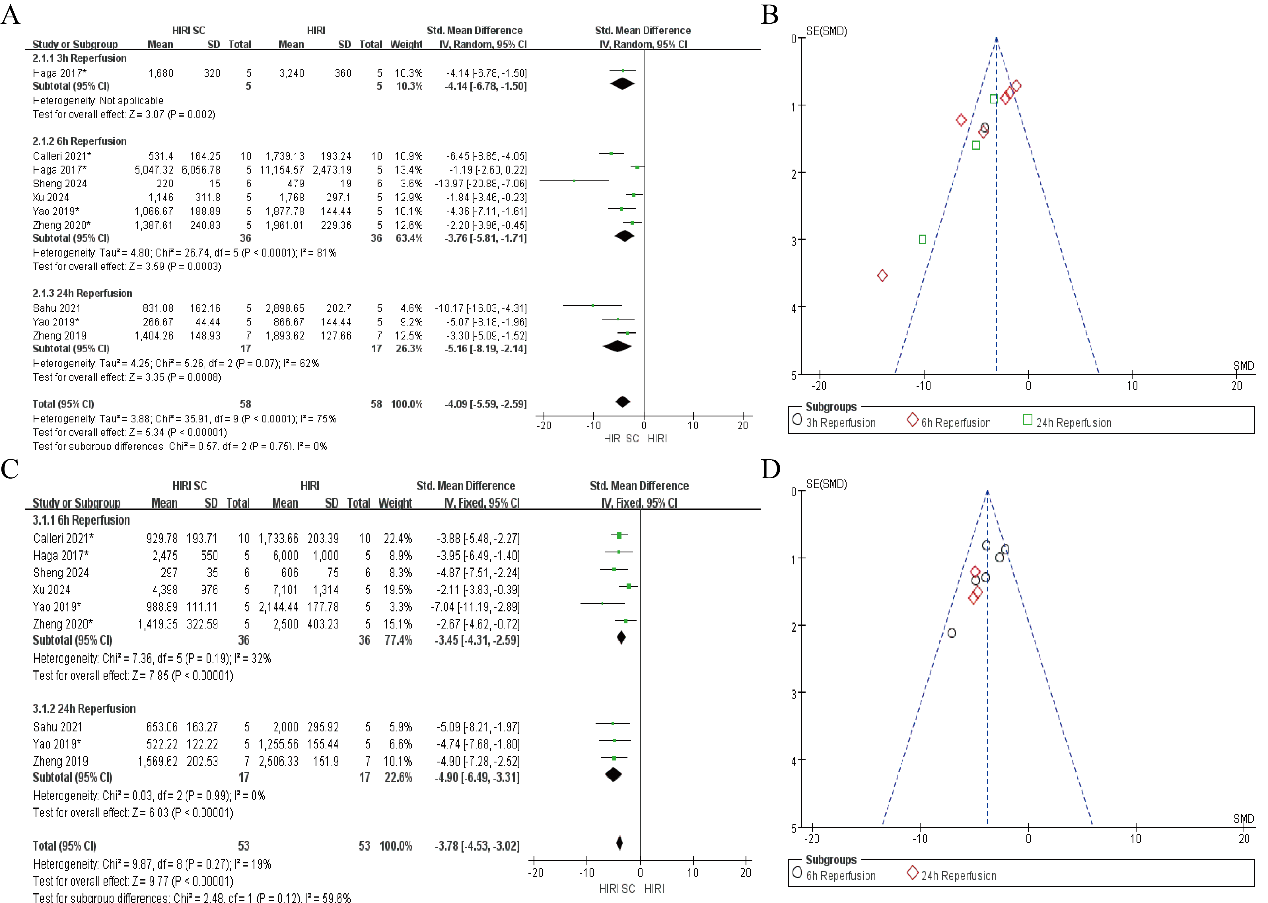


**Supplementary Figure S2.** Forest and funnel plots depicting the effects of stem cell-based interventions on liver function markers and publication bias in rodent models subjected to 70% HIRI with 90 minutes of ischemia. ALT levels were significantly reduced (10 studies; SMD=4.09, 95% CI: 2.59-5.59; *p* < 0.00001) (A), and AST levels were significantly reduced (9 studies; SMD = 3.78, 95% CI: 3.02-4.53; *p* < 0.00001) (C). Funnel plots showed no evidence of publication bias in the meta-analyses of ALT (B) and AST (D). *Studies using exosomes/vesicles or their modified forms.

**Supplementary Table S1. Search strategy**

| **Databases** | **Search items** |
| --- | --- |
| PubMed | #1 Mice[MeSH] OR rat[MeSH] OR murinae[MeSH] OR mus[tiab] OR mouse[tiab] OR rats[tiab] OR rattus[tiab]  #2 Liver[MeSH] OR livers[tiab] OR hepat*[tiab]  #3 stem cells[MeSH] OR Stem Cell[tiab] OR Mother Cell[tiab] OR Mother Cells[tiab] OR Progenitor Cell[tiab] OR Progenitor Cells[tiab] OR Colony-Forming Unit[tiab] OR Colony-Forming Units[tiab]  #4 Ischemia Reperfusion Injury[MeSH] OR Reperfusion Injuries[tiab] OR Injury, Ischemia Reperfusion[tiab] OR Ischemia Reperfusion Injuries[tiab] OR Injury, Reperfusion[tiab] OR Reperfusion Damage[tiab] OR Damage, Reperfusion[tiab] OR Reperfusion Damages[tiab] OR IRI[tiab]  #5 #1 AND #2 AND #3 AND #4 |
| Web of Science | #1 ((((((TS=(Mice)) OR TS=(mus)) OR TS=(mouse)) OR TS=(murinae)) OR TS=(rat)) OR TS=(rats)) OR TS=(rattus)  #2 ((TS=(liver)) OR TS=(livers)) OR TS=(hepat*)  #3 (((((((TS=(stem cells)) OR TS=(Stem Cell)) OR TS=(Mother Cell)) OR TS=(Mother Cells)) OR TS=(Progenitor Cell)) OR TS=(Progenitor Cells)) OR TS=(Colony-Forming Unit)) OR TS=(Colony-Forming Units)  #4 ((((((((TS=(Ischemia Reperfusion Injury)) OR TS=(Reperfusion Injuries)) OR TS=(Injury, Ischemia Reperfusion)) OR TS=(Ischemia Reperfusion Injuries)) OR TS=(Injury, Reperfusion)) OR TS=(Reperfusion Damage)) OR TS=(Damage, Reperfusion)) OR TS=(Reperfusion Damages)) OR TS=(IRI)  #5 #1 AND #2 AND #3 AND #4 |
| Embase | #1 'mouse'/exp  #2 'mice'/exp OR mice OR (mus:ab,ti AND genus:ab,ti) OR 'newborn mice':ab,ti OR mouse:ab,ti  #3 #1 OR #2  #4 'rat'/exp  #5 rats:ab,ti OR rattus:ab,ti OR rat:ab,ti  #6 #4 OR #5  #7 #3 OR #6  #8 'liver'/exp  #9 hepar:ab,ti OR 'hepatic gland':ab,ti OR 'hepatic glandular system':ab,ti OR 'hepatic organ':ab,ti OR 'hepatic system':ab,ti OR 'human liver':ab,ti OR liver:ab,ti  #10 #8 OR #9  #11 'stem cell'/exp  #12 'cell, stem':ab,ti OR 'precursor cell':ab,ti OR 'progenitor cell':ab,ti OR 'stem cells':ab,ti OR 'stem cell':ab,ti  #13 #11 OR #12  #14 'reperfusion injury'/exp  #15 'injury, reperfusion':ab,ti OR ('ir injury':ab,ti AND reperfusion:ab,ti) OR (iri:ab,ti AND 'ischemia reperfusion injury':ab,ti) OR 'ischaemia reperfusion injury':ab,ti OR ('ischaemia/reperfusion':ab,ti AND ir:ab,ti AND injury:ab,ti) OR ('ischaemic reperfusion':ab,ti AND ir:ab,ti AND injury:ab,ti) OR 'ischaemic reperfusion injury':ab,ti OR 'ischemia reperfusion injury':ab,ti OR ('ischemia/reperfusion':ab,ti AND ir:ab,ti AND injury:ab,ti) OR ('ischemic reperfusion':ab,ti AND ir:ab,ti AND injury:ab,ti) OR 'ischemic reperfusion injury':ab,ti OR 'reperfusion cell injury':ab,ti OR 'reperfusion organ injury':ab,ti OR 'reperfusion tissue injury':ab,ti OR 'reperfusion-associated injury':ab,ti OR 'reperfusion-induced injury':ab,ti OR 'reperfusion-induced organ injury':ab,ti OR 'reperfusion-induced tissue injury':ab,ti OR 'reperfusion-related injury':ab,ti OR 'reperfusion injury':ab,ti  #16 #14 OR #15  #17 #7 AND #10 AND #13 AND #16 |
| Cochrane Library | #1 MeSH descriptor: [Mice] explode all trees  #2 MeSH descriptor: [Rats] explode all trees  #3 MeSH descriptor: [Murinae] explode all trees  #4 ("Mus"):ti,ab,kw AND ("Rattus"):ti,ab,kw  #5 #1 OR #2 OR #3 OR #4  #6 MeSH descriptor: [Liver] explode all trees  #7 (livers):ti,ab,kw OR (hepat*):ti,ab,kw  #8 #6 OR #7  #9 MeSH descriptor: [Stem Cells] explode all trees  #10 ("stem cell"):ti,ab,kw OR ("mother cell"):ti,ab,kw OR (Mother Cells):ti,ab,kw OR ("progenitor cell support"):ti,ab,kw OR ("colony forming unit"):ti,ab,kw  #11 #9 OR #10  #12 MeSH descriptor: [Reperfusion Injury] explode all trees  #13 ("reperfusion injuries"):ti,ab,kw OR (Injury, Ischemia Reperfusion):ti,ab,kw OR (Ischemia Reperfusion Injuries):ti,ab,kw OR (Injury, Reperfusion):ti,ab,kw OR (Reperfusion Damage):ti,ab,kw  #14 #12 OR #13  #15 #5 AND #8 AND #11 AND #14 |
| ClinicalTrials.gov | Mice OR rat OR murinae OR mus OR mouse OR rats OR rattus AND Liver AND Ischemia Reperfusion Injury OR Reperfusion Injuries OR Injury, Ischemia Reperfusion OR Ischemia Reperfusion Injuries OR Injury, Reperfusion OR Reperfusion Damage AND stem cell OR Mother Cell |

| **Supplementary Table S2. Summary of the Mechanisms and Outcome Data of Stem Cell Therapy for HIRI** | | | | | | |
| --- | --- | --- | --- | --- | --- | --- |
| **Author** | **Outcome index** | | | | | **mechanism** |
|  | **Effect on HIRI** | **Biochemical indicators of liver function** | **Histopathology** | **Oxidative stress and inflammation regulation** | **Cell apoptosis and regeneration** |  |
| **Stem Cells** |  |  |  |  |  |  |
| Xu C et al.2024^[66]^ | alleviate | decrease ALT and AST | necrotic areas were smaller with preserved cellular structure | activating autophagy through upregulation of IFNγ | Cleaved-Caspase3 decreased and Bcl-2 increased in hMSCs | HMSCs attenuate fatty liver IRI by IFNγ-mediated MAPK activation to induce hepatoprotective autophagy. |
| Shang LC et al.2023^[59]^ | protect | NE | decrease liver necrosis | inhibit mitochondrial fission, attenuated infammatory responses | NE | MSCs facilitated M1-M2 phenotypic polarization through inhibiting Drp-1 dependent mitochondrial fssion and further attenuated HIRI. |
| Kartal B et al.2023^[44]^ | protect | NE | reduce portal inflammation, hepatocyte edema, cytoplasm deformation and coagulation | NE | NE | ADSCs decreased excessive autophagy, hepatocyte damage, and liver pathology following HIRI. |
| Chen K et al.2022^[43]^ | prevent | reduce AST and ALT | less cell destruction and vacuolization | reduce hepatocytes Hypoxia/Reoxygenation injury and ROS damage in co-culture | ameliorate cell survival in 120-Min HIRI | ASCL Prevents HIRI in Rats by Inhibiting Inflammasome Activation |
| Khosravi-Farsani S et al.2022^[79]^ | treat | decrease ALT, AST, and LDH | decrease edema and tissue necrosis | NE | NE | NE |
| Piao C et al.2021^[55]^ | treat | decrease ALT, AST, and ALP | NE | mitigate inflammation and oxidative stress | NE | Post-surgical ADSCs transplantation accelerated liver repair by activating Keap1/Nrf2, reducing inflammation and oxidative stress to restore the hepatic microenvironment. |
| Zheng J et al.2020^[6]^ | alleviate | reduce ALT, AST and LDH | reduction in Suzuki’s score | attenuate oxidative stress, upregulated PINK1-dependent mitophagy | decrease the expression of caspase-3 and apoptosis-related protein, TUNEL assay | MSCs ameliorate hepatocellular apoptosis mediated by PINK1-dependent mitophagy in HIRI through AMPKα activation. |
| Li C et al.2019^[63]^ | alleviate | decrease ALT | preserved hepatic architecture, with minimal sinusoidal congestion and without edema, vacuolization, or necrosis | inhibits proinflammatory mediators (reduced TNF-α and IL-1β) | NE | MSCs Regulate HIPPO Signaling and β-Catenin activation and control macrophage polarization in IR-stressed livers. |
| Zare MA et al.2019^[47]^ | protect | decrease ALT and AST | decrease congestion, hepatocyte vacuolization, necrosis, and inflammatory cell infiltration | NE | increase antiapoptotic Bcl2 expression and reduce proapoptotic Bax expression | BM-MSCs protect against HIRI by downregulating miR-370, which modulates the Bcl-2/BAX pathway to attenuate liver damage. |
| Wang X et al.2018^[37]^ | protect | decrease ALT and AST | reduce hepatocellular necrosis and cytoplasmic vacuolization | NE | NE | BM-MSCs protected against HIRI via the promotion of HO-1-mediated autophagy. |
| Qi X et al.2018^[61]^ | alleviate | NE | NE | suppress the inflammatory response | suppress hepatic apoptosis and promoted liver regeneration | Engineered hiPSC-MSCs delivering GPx3 ameliorated HIRI via inhibition of hepatic senescence. |
| Li S et al.2018^[46]^ | alleviate | decrease ALT and AST | alleviate portal inflammation, hepatocyte swelling, cytoplasm rarefaction, and coagulative necrosis | reduce neutrophil infiltration, attenuate neutrophil recruitment | NE | MSCs Ameliorate HIRI via Inhibition of Neutrophil Recruitment. |
| Isbambetov A et al.2016^[68]^ | Equal distribution | NE | NE | NE | promote regeneration | After HIRI, MSCs migrate equally to ischemic and nonischemic regions, likely driven by regenerative signals. |
| Saat T C et al.2016^[64]^ | NE | decrease ALT and AST | NE | IL-6 levels were significantly lower 48h post-reperfusion. | NE | NE |
| Wang X et al.2016^[72]^ | improve | decrease ALT, AST, and MDA | decrease hepatocyte degeneration, necrosis, and fibrosis | NE | upregulation of hepatocyte growth factor (HGF) and downregulation of α-SMA | HAD-MSCs transplantation improves liver function in rats, likely by regulating HGF and α-SMA expression, promoting repair of HIRI. |
| Nowacki M et al.2015^[73]^ | NE | NE | NE | NE | NE | NE |
| Lee SC et al.2015^[48]^ | protect | decrease ALT and AST | decrease hepatocellular necrosis, congestion, and cytoplasmic vacuolization | IL-6 levels were decreased, decreased neutrophil infiltration into the liver | NE | ASC and ASC-secretome infusions alleviated liver damage and improved the microenvironment after HIRI, likely through paracrine effects mediated by the ASC secretome. |
| Fouraschen SM et al.2015^[42]^ | treat | decrease ALT and AST | NE | downregulate the inflammatory genes TNFA and IL1RN | stimulate hepatocyte proliferation | MSC-derived factors aid tissue regeneration in small-for-size livers under ischemic conditions but do not prevent early IRI. |
| Saidi R F et al.2014^[49]^ | alleviate | decrease ALT and IL-6 | less congestion, vacuolization, and necrosis scores | NE | There were more PCNA-positive cells | HADMSCs attenuated liver injury and increased regeneration in IRI/PH models, representing a potential therapeutic strategy to IRI and liver transplantation. |
| Saito Y et al.2013^[69]^ | alleviate | decrease AST, ALT, and T-Bil | NE | NE | stimulate liver regeneration | ADSCs protect hepatocytes and promote liver regeneration in hepatectomy/IRI models through mechanisms independent of VEGF signaling. |
| Sun CK et al.2012^[7]^ | alleviate | NE | NE | anti-inflammatory and an antioxidative | suppress the number of apoptotic nuclei | Systemic administration of autologous ADMSCs alleviates HIRI in rats by preserving hepatocytes and reducing inflammation, oxidative stress, and apoptosis. |
| Seki T et al.2012^[38]^ | treat | NE | NE | NE | elevate mitotic index, PCNA levels, and other regeneration-associated proteins in the liver | ADSCs transplantation promotes hepatic regeneration after HIRI and subsequent hepatectomy in rats. |
| Pan GZ et al.2012^[70]^ | alleviate | decrease AST, ALT, and LDH | decrease hepatocellular necrosis | NE | promote hepatic regeneration | Bone marrow MSCs ameliorate HIRI via inactivation of the MEK/ERK signaling pathway in rats |
| **Stem Cells Modification** |  |  |  |  |  |  |
| Chen H et al.2024^[34]^ | alleviate | reduce AST, ALT, and LDH | mitigate hepatocytes necrosis | restore Mitochondrial Dysfunction, inflammatory chemotaxis (TNF-α) | reduce cellular apoptosis | MMCLs attenuated HIRI by targeting liver lesions, suppressing ROS, inhibiting mitochondrial fission, and reducing apoptosis. |
| Sheng MW et al.2024^[12]^ | alleviate | decrease AST, ALT, and LDH | decrease hepatocellular necrosis, vacuolation and bleb formation | NE | promote hepatocyte proliferation and Inhibited hepatocyte apoptosis | CD47-overexpressed MSCs can alleviate fatty liver I/R injury in mice, and activation of SIRPa and subsequent inhibition of pyroptosis are involved in the process. |
| Ko SF et al.2023^[19]^ | protect | NE | NE | suppressing the inflammation and innate and adaptive immune responses | decreased expressions of mitochondrial Bax, cleaved-caspase 3 and cleaved PARP | Tacrolimus combined with ADMSCs inhibits acute HIRI-induced inflammatory and immune responses, protecting liver parenchyma. |
| Zhang Q et al.2023^[26]^ | treat | decrease ALT and AST | decrease inflammatory infiltration, decreased Suzuki’s injury scores | N/A | attenuate I/R-induced hepatocyte apoptosis | IFN-γ-primed MenSCs improved HIRI treatment via IDO-mediated AMPK-mTOR-autophagy activation and Treg-dependent immune tolerance. |
| Tian X et al.2023^[11]^ | protect | reduce the ALT, AST, TBIL, and LDH | NE | reduce the IL-1β, IL-6 and TNF-α | decrease hepatocyte apoptosis | HO-1/BMMSCs protected against severe steatotic liver IRI by inhibiting ferroptosis through the AMPK–Nrf2–FTH1 pathway. |
| Owen A et al.2022^[18]^ | alleviate | reduce ALT and ALP | NE | suppress lymphocytes and secrete IL-10 and osteoprotegerin | NE | PDGFRα/Sca-1 Sorted MSC Reduce Liver Injury in Murine Models of HIRI. |
| Lin Y et al.2022^[23]^ | alleviate | NE | attenuate inflammatory infiltration and hepatocyte necrosis | suppress inflammation and oxidative stress in liver tissues | restrained apoptosis in Liver Tissues | Baicalin combined with BMSCs outperformed monotherapy in HIRI treatment, likely via HO-1 activation and NF-κB suppression. |
| Sahu A et al.2021^[33]^ | treat | reduce ALT and AST | little congestion, vacuolization, and necrotic hepatocytes | attenuate oxidative stress, enhance anti-inflammatory properties | inhibit apoptosis | The nanozyme impregnation MSCs attenuated HIRI via oxidative resistance and enhanced paracrine/anti-inflammatory activity. |
| Li Q et al.2021^[13]^ | alleviate | decrease the ALT and AST | improve sinusoidal congestion, cytoplasmic vacuolization and necrosis | attenuate the oxidative stress response | decrease or inhibit cell apoptosis (Bcl‑2, Bax and caspase‑3) | SOD2‑overexpressing BM‑MSCs may provide therapeutic support in HIRI by inhibiting oxidative stress and hepatocyte apoptosis. |
| Tan Y et al.2021^[56]^ | treat | decrease ALT and T-Bil | NE | promote the anti-inflammatory, anti-oxidant | enhance the functions of cell homing and repair | BMSCs repair oxidative stress injury and fatty liver IRI, while TNF-α-stimulated BMSCs enhance tissue and cell repair. |
| Zheng J et al.2019^[20]^ | alleviate | NE | NE | attenuate the inflammatory response and reduces oxidative stress | NE | Preconditioning of UC-MSCs by rapamycin increases cell migration and ameliorates HIRI in mice via the CXCR4/CXCL12 axis. |
| Liu J et al.2019^[28]^ | protect | decrease ALT, AST, and LDH | NE | alleviate oxidative stress | Down-regulate hepatocyte apoptosis | Mild hypothermia combined with ADMSCs transplantation protects against HIRI, likely by activating the ERK pathway to reduce hepatocyte apoptosis in vivo. |
| Sun Y et al.2018^[29]^ | treat | decrease ALT and AST | reduce the hepatic necrosis and infammation | reduce the production of pro-inflammatory chemokines | did not promote hepatic regeneration but inhibit apoptosis. | 3D UC-MSCs reduced HIRI in rats by lowering aminotransferase, liver damage scores, neutrophil infiltration, hepatocyte apoptosis, and inflammation-related gene. |
| Feng J et al.2018^[24]^ | alleviate | decrease LDH, AST, and ALT | Alleviate HIRI-induced hepatic sinusoid congestion, hepatocyte vacuolization, and lobular disorder | NE | NE | Dexmedetomidine and midazolam enhanced hBM-MSC hepatoprotection in HIRI via receptor binding, paracrine modulation, and NF-κB p65 regulation, outperforming propofol. |
| Qiao PF et al.2015^[30]^ | NE | decrease ALT and AST | alleviation of histopathological damage | NE | NE | HSP induces autophagy following exposure to H2O2 via the p38MAPK/mTOR pathway, which enhanced MSC survival and improved MSC repair following HIRI in rats. |
| Fu J et al.2014^[16]^ | protect | decrease AST, ALT, and LDH | reduce portal inflammation, cytoplasmic vacuolation, apoptotic body production, and hepatocellular necrosis | NE | inhibite hepatocyte apoptosis, promote hepatic regeneration | MSC transplantation provides support to the HIRI by inhibiting hepatocellular apoptosis and stimulating NAT8 regeneration. |
| **Exosomes/Vesicles** |  |  |  |  |  |  |
| Li H et al.2024^[51]^ | aggravate | suppress elevation of ALT and AST | reduce loss of hepatocyte integrity, extensive hepatocyte necrosis, and inflammatory infiltration | NE | decrease apoptosis-associated proteins (CC-3 and Bax protein) | BMMSCs Extracellular Vesicle-derived miR-27b-3p activates the Wnt/Β-catenin Pathway by Targeting SMAD4 and Aggravates HIRI. |
| Li H et al.2023^[52]^ | alleviate | decrease ALT/AST | reduce hepatocyte necrosis and improved cellular integrity | NE | decrease hypoxia‑induced apoptosis and improved cell viability | BMSC‑exosomes miR‑25‑3p Regulates the p53 Signaling Pathway Through PTEN to Inhibit Cell Apoptosis and Ameliorate HIRI. |
| Gong Y et al.2023^[71]^ | alleviate | reduce the AST, ALT, and LDH | NE | NE | enhance proliferation and inhibit apoptosis | Exosomes derived from human ADSCs alleviate HIRI through the miR-183/ALOX5 axis |
| Zhang Y et al.2022^[60]^ | protect | reduce AST, ALT and LDH | reduce liver necrosis and apoptosis, with slight swelling of nuclei and endoplasmic reticulum | reduce oxidative stress and lipid peroxidation | NE | Attenuation of HIRI by ADSCs‑exo treatment via ERK1/2 and GSK‑3β signaling pathways |
| Piao C et al.2022^[62]^ | treat | NE | inhibits the pyroptosis resulting from inflammatory responses | inhibits the pyroptosis resulting from inflammatory responses | reduces liver injury, and promotes liver regeneration | ADSCs-Exo mitigates HIRI by the inhibition of the NF-κB pathway and the activation of the Wnt/β catenin pathway. |
| Zhang Q et al.2021^[45]^ | protect | reduce ALT, AST, TBIL, ALP, and LDH | alleviate ultrastructural damage with nuclear shrinkage, increase number of mitochondria | reduces oxidative stress, inhibits excessive mitochondrial fission after I30R+PH | inhibit apoptosis due to I30R+PH | ADSCs-exo protects against HIRI subsequent to hepatectomy by reducing mitochondrial fission, promoting mitochondrial fusion and improving mitochondrial biogenesis. |
| Calleri A et al.2021^[50]^ | protect | reduce ALT and LDH | reduce large areas of vascular congestion, cell vacuolization and hydropic degeneration | reduce TNF-α, CXCL-10, CCL-2 | NE | 3×109 HLSC-EV can modulate HIRI by preserving tissue integrity and by reducing transaminases release and inflammatory cytokines expression. |
| Zheng J et al.2020^[36]^ | prevent | decrease ALT, AST, and LDH | reduce disorder of hepatic lobules, tissue necrosis, and infiltration of inflammatory cells | suppress the levels of IFN-𝛾, IL-6, and TNF-𝛼 | decrease hepatocellular apoptosis | UC-MSC-EVs mitigate HIRI inflammation by delivering CCT2, which suppresses CD4+ T cell activation via the Ca²⁺-calcineurin-NFAT1-CD154 axis. |
| Xie K et al.2019^[65]^ | alleviate | abolish increase of ALT and AST | NE | the balance between Treg and Th17 cells | NE | Exosomal miR-1246 derived from hUCB-MSCs attenuates HIRI by modulating T helper 17/regulatory T balance. |
| Xie K et al.2019^[67]^ | improve | decrease ALT and AST | NE | NE | reduce apoptotic cells | Exosomes derived from hUCB-MSCs improve HIRI via delivering miR-1246. |
| Nong K et al.2019^[54]^ | alleviate | NE | reduce hydropic degeneration, necrosis, inflammation, and hepatic sinusoid swelling in liver cells | reduce the occurrence of the inflammatory response | reduce the occurrence of the apoptosis | Overexpression of miR-148a in MSC-exos inhibits CaMKII and TLR4 expression in I/R tissues, reducing inflammation and apoptosis. |
| Yao J et al.2019^[57]^ | alleviate | decrease ALT, AST, and ALP | inhibit hepatocyte necrosis | reduce hepatic inflammation, neutrophil infiltration, and oxidative stress of hepatic tissue | prevent hepatocytes from oxidative stress-induced cell death | HucMSC-EVs mitigate HIRI by decreasing neutrophil infiltration and oxidative stress in vivo, and suppressing neutrophil respiratory burst and hepatocyte death in vitro. |
| Anger F et al.2019^[39]^ | improve | reduce ALT and AST | reduce hepatic necrosis | change the intrahepatic transcription of inflammation-associated genes after hepatic IRI | promote early hepatocyte proliferation after hepatic IRI | HMSCs-EV promote liver repair after HIRI by lowering injury markers, suppressing inflammation, and stimulating hepatocyte proliferation. |
| Du Y et al.2017^[40]^ | protect | decrease ALT and AST | suppress hepatocyte necrosis and sinusoidal congestion | NE | promote hepatocyte proliferation | HiPSC-MSCs-Exo could alleviate HIRI via activating sphingosine kinase and sphingosine-1-phosphate pathway in hepatocytes and promote cell proliferation. |
| Nong K et al.2017^[41]^ | protect | decrease ALT and AST | suppress hepatocyte necrosis and sinusoidal congestion | decrease the inflammatory response, alleviated oxidative stress | inhibit the apoptotic response | HiPSC-MSCs-Exo alleviate HIRI, possibly via suppression of inflammatory responses, attenuation of the oxidative stress response and inhibition of apoptosis. |
| Haga H et al.2017^[58]^ | protect | decrease ALT, AST, ALP, and BUN | reduce widespread necrosis | modulate oxidative stress and dampen inflammatory responses | reduce number of caspase-3 positive cells | The administration of extracellular vesicles derived from BMMSCs may ameliorate HIRI by reducing hepatic injury through modulation of the inflammatory response. |
| **Exosomes/Vesicles Modification** | |  |  |  |  |  |
| Zhang B et al.2024^[21]^ | alleviate | suppress elevation of ALT and AST | NE | reduce inflammatory, ameliorate Th17/Treg imbalance mediators | NE | Ba-Exo mitigates HIRI by inducing FGF21 expression in liver cells, inhibiting JAK2/STAT3, and activating FOXO1. |
| Piao C et al.2024^[27]^ | treat | reduce AST, LDH, and TBIL | NE | alleviate HIR-induced mitochondrial damage and oxidative stress response | NE | Met-Exo promote mitochondrial biosynthesis and fusion-related protein expression after partial resection of hepatic IRI by regulating AMPK/SIRT1 signaling pathway. |
| Li R et al.2024^[31]^ | alleviate | reduce AST and ALT | diminished HIRI-mediated fibrosis | alleviate inflammation responses (IFN-γ, TNF-α and IL-6) | alleviate hepatocellular apoptosis (Caspase-3, Bax, and Bad) | Phosphatidylserine (PS) on injured hepatocytes helps MSC-EVs bind via MFGE8, enabling their therapeutic effect on HIRI. |
| Miao L et al.2024^[15]^ | protect | decrease ALT and AST | reduce loss of hepatocyte integrity, extensive hepatocyte necrosis, and inflammatory response | alleviate inflammatory response | attenuate hepatic apoptosis | Extracellular vesicles containing GAS6 protect the liver from IRI by enhancing macrophage efferocytosis via MerTK-ERK-COX2 signaling |
| Sameri MJ et al.2022^[35]^ | protect | reduce AST and ALT | reduce hepatocyte damage, congestion and necrosis, reduced TNF-a and IL-6 | revert the total oxidant status (TOS) in HIRI | decrease BAX, BAK1, Bcl2, MALAT1, cleaved caspase-3, and TUNEL-positive cells | NaHS-preconditioned exosomes (MSC-Exo and H2S-Exo) demonstrated hepatoprotective effects against HIRI in mice by attenuating inflammation, oxidative stress, and apoptosis. |
| Li X et al.2022^[14]^ | alleviate | reduce the ALT and AST | reduce the liver histopathological injury and hepatocyte death, reduced the Suzuki score | reduce proinflammatory cytokines (IL-6, TNF-α, and IL-1β) | reduce the percentage of TUNEL positive staining | HO-1/BMMSCs could suppress ferroptosis by targeting Ireb2 via the exosomal transfer of miR-29a-3p to alleviate steatotic HIRI in Rats. |
| Zhang L et al.2020^[17]^ | alleviate | decrease ALT and AST | NE | NE | regulate apoptosis and autophagy by targeting FAS, Caspase-3, and related genes in I/R | UC-MSC exosomal miR-20a protects against HIRI by regulating apoptosis- and autophagy-related genes, including Caspase-3, mTOR, P62, and LC3II. |
| Yang B et al.2020^[32]^ | alleviate | decrease ALT and AST | reduce hemorrhage, necrosis and the structure of the liver lobe damaged | enhance autophagy, inhibiting excessive ROS | reduce hepatocyte apoptosis | MSC‐Heps‐Exo Reduce HIRI by Enhancing Autophagy |
| Wei X et al.2020^[22]^ | treat | decrease ALT and AST | NE | inhibit hypoxia-induced c-CASP-3, c-PARP, NOX1, NOX2, and LPS-induced TLR4, HMGB1, IL-1β, TNF-α | NE | GA enhanced MSC-exosome efficacy in I/R rats by stabilizing peripheral blood cells, lowering ALT/AST, and normalizing inflammatory proteins. |

HIRI, Hepatic ischemia-reperfusion injury; SC, stem cells; HSP, Heat shock pretreatment; NE, not evaluated
